# A high-quality genome assembly of the tetraploid *Teucrium chamaedrys* unveils a recent whole-genome duplication and a large biosynthetic gene cluster for diterpenoid metabolism

**DOI:** 10.1016/j.xplc.2025.101393

**Published:** 2025-06-03

**Authors:** Abigail E. Bryson, Kevin L. Childs, Nicholas Schlecht, Davis Mathieu, John P. Hamilton, Haoyang Xin, Jiming Jiang, C. Robin Buell, Bjӧrn Hamberger

**Affiliations:** 1 Department of Biochemistry, Michigan State University, East Lansing, MI 48823, USA; 2 Department of Plant Biology, Michigan State University, East Lansing, MI 48823, USA; 3 Department of Horticulture, Michigan State University, East Lansing, MI 48823, USA; 4 Center for Applied Genetic Technology, University of Georgia, Athens, GA 30602, USA; 5 Department of Crop & Soil Sciences, University of Georgia, Athens, GA 30602, USA; 6 Institute of Plant Breeding, Genetics, & Genomics, University of Georgia, Athens, GA 30602, USA; 7 The Plant Center, University of Georgia, Athens, GA 30602, USA

**Keywords:** Lamiaceae (mint), *Teucrium*, diterpenoid, biosynthetic gene cluster, BGC

## Abstract

*Teucrium chamaedrys*, commonly known as wall germander, is a small woody shrub native to the Mediterranean region. Its name is derived from the Greek words meaning “ground oak,” as its tiny leaves resemble those of an oak tree. *Teucrium* species are prolific producers of diterpenes, endowing them with valuable properties widely utilized in traditional and modern medicine. Sequencing and assembly of the 3-Gbp tetraploid *T. chamaedrys* genome revealed 74 diterpene synthase genes, with a substantial number of these genes clustered at four synteny genomic loci, each harboring a copy of a large diterpene biosynthetic gene cluster. Comparative genomics revealed that this cluster is conserved in the closely related species *Teucrium marum*. Along with the presence of several cytochrome p450 sequences, this region is among the largest biosynthetic gene clusters identified. *Teucrium* is well known for accumulating clerodane-type diterpenoids, which are produced from a kolavenyl diphosphate precursor. To elucidate the complex biosynthetic pathways of these medicinal compounds, we identified and functionally characterized several kolavenyl diphosphate synthases from *T. chamaedrys*. The remarkable chemical diversity and tetraploid nature of *T. chamaedrys* make it a valuable model for studying genomic evolution and adaptation in plants.

## Teaser

The mint family holds one of the largest repositories of chemical diversity in terpene bioproducts. This study reports the genome assembly and annotation for *Teucrium chamaedrys*, a member of the germander genus with a recently duplicated genome. Comparative genomics reveals mechanisms involving biosynthetic gene clusters in driving the dynamic evolution and proliferation of complex pathways of specialized metabolism.

## Introduction

The Lamiaceae (mint) family includes culturally and economically important plants such as peppermint, lavender, sage, rosemary, and teak. It is the third-largest family of flowering plants, with an estimated 7000 species. However, representative genomes are limited, with only about 0.66% (46) published to date. Sampling understudied clades in the Lamiaceae can help elucidate the basis of specialized metabolism, as this family is known to produce nearly 7500 unique plant natural products relevant to human health and industry (Dictionary of Natural Products 30.2). The subfamily Ajugoideae (syn. Teucrioideae) is one such understudied clade, which includes approximately 770 species and only three published genomes (Ritz et al., 2023; Smit et al., 2024). Within Ajugoideae, the polyphyletic *Teucrium* is one of the largest genera, with approximately 300 species. *Teucrium* has been used for millennia, with historical applications such as treating asthma in ancient Greece (Menichini et al., 2009). *Teucrium* species are also well known for their insect antifeedant activity; allelopathic inhibition of cosmopolitan weeds; antimicrobial, antiviral, anti-inflammatory, and hepatotoxic effects; and potential as a selective anticancer agent for colorectal cancer (Klein Gebbinck et al., 2002; Milutinović et al., 2019; Candela et al., 2020). *Teucrium chamaedrys*, or wall germander, is a woody shrub native to the Mediterranean region and is one of the most frequently cited *Teucrium* species in folk medicine (Jarić et al., 2020). It is specifically recognized in ethnobotanical studies for treating a wide variety of health issues, including digestive disorders, hypertension, and malaria (Pieroni et al., 2004; di Tizio et al., 2012; Arı et al., 2015; Jarić et al., 2020).

**Figure 1:**
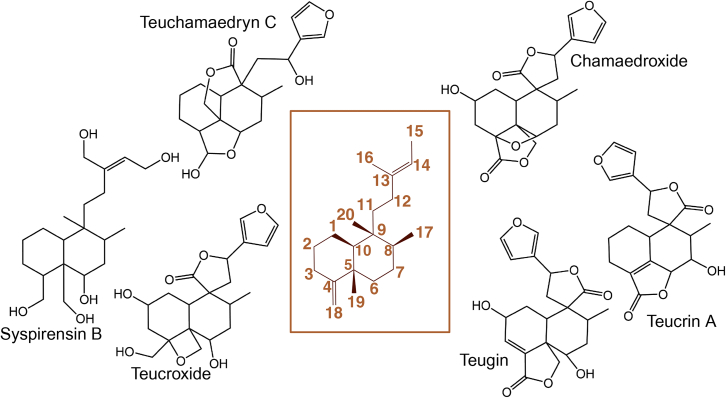
Clerodane skeleton and select clerodanes from *T. chamaedrys*. *Teucrium*, specifically *T. chamaedrys*, is rich in clerodane-type diterpenoids. Middle box features numbered carbons on a typical clerodane skeleton.

The medicinal properties of plants are typically a consequence of their specialized metabolite profiles. Ajugoideae—*Teucrium* in particular—is well known for its abundance of diterpenoids (Dictionary of Natural Products 30.2). Generally, diterpenoids are formed by the sequential activity of two diterpene synthases (diTPSs). A class II diTPS (TPS-c) first catalyzes the proton-mediated cyclization of a 20-carbon isoprenoid diphosphate, usually geranylgeranyl diphosphate (GGDP). Then, a class I (often a TPS-e) diTPS cleaves the diphosphate, further modifying the diterpene structure. *Teucrium* is especially rich in clerodane-type diterpenoids (Li et al., 2016; Schlecht et al., 2024). Clerodane synthases typically generate a class II product, either (−)-kolavenyl diphosphate ((−)-KDP), iso-KDP, or more rarely, *cis*-*trans*-clerodienyl diphosphate. To date, characterized iso-KDP synthases have only been identified in Lamiaceae species, including *Ajuga reptans*, *Scutellaria barbata,* and *Scutellaria baicalensis* (Johnson et al., 2019; Qiu et al., 2023). (−)-KDP synthases have been characterized in *Salvia divinorum*, *Salvia splendens*, *Vitex agnus-castus*, *Callicarpa americana*, *S. barbata*, *S. baicalensis*, and *Tripterygium wilfordii* (Andersen-Ranberg et al., 2016; Hansen et al., 2017; Pelot et al., 2017; Heskes et al., 2018; Hamilton et al., 2020). Iso-KDP differs from (−)-KDP by the site of final deprotonation, placing the double bond along the 4,18 bond rather than the 3,4 bond (Figure 1). The third and most uncommon structure has exclusively been found in the monocot species *Panicum virgatum*. It is a *cis*-*trans*-clerodienyl diphosphate, which, while sharing the same final quenching as (−)-KDP, is a different stereoisomer (Pelot et al., 2018). A variety of clerodane-derived products have been characterized specifically from *T. chamaedrys*, including various neo-clerodanes, chamaedryosides A–C, Teucrin, and others (Figure 1; Bedir et al., 2003; Fiorentino et al., 2009; Sadeghi et al., 2022; Dictionary of Natural Products 30.2).

The diversity of plant natural products is often driven by gene duplication via several mechanisms. Duplications can significantly increase novel gene formation by dispersing selective pressure, thereby allowing an explosion of metabolic diversity (Ren et al., 2018). Such duplications can occur through tandem or segmental duplication, both of which copy a region locally and can be the result of unequal DNA crossover events (Achaz et al., 2000). Repeats can also be introduced via retrotransposition, recognizable by the lack of introns and the presence of nearby inverted repeats (Hughes et al., 2003; Field et al., 2011). However, the most radical duplication method is whole-genome duplication (WGD). It is estimated that around 35% of all extant angiosperm species are polyploids with a history of WGDs (Wood et al., 2009; Landis et al., 2018; Godden et al., 2019). Nearly two-thirds (65%) of annotated plant genes are duplicated, with most derived from WGD events (Panchy et al., 2016).

To better understand how polyploidy affects diTPSs and chemical diversity in *Teucrium*, we sequenced and assembled the large (3 Gbp) tetraploid genome of *T. chamaedrys.* A recent WGD has quadrupled TPSs in *T. chamaedrys*, including four copies of a large gene cluster containing the majority of the diTPSs. This cluster is also conserved in the closely related species *Teucrium marum* and predates the WGD, with diterpene chemistries in characterized species distinct from the clerodanes (Smit et al., 2024). The physical clustering of these diTPSs creates one of the largest biosynthetic gene clusters (BGCs) to date, spanning around 500 Kbp, within the range of the the 2 Mbp cluster present in *Ginkgo biloba* and the 580 Kbp cluster in opium poppy (Forman et al., 2022; Guo et al., 2018). Since *Teucrium* species are well known for their clerodane-derived products, we functionally characterized all four putative clerodane synthases in *T. chamaedrys*, along with a representative synthase from *Teucrium canadense*. Using comparative genomic, phylogenetic, and biochemical methods, we present the genetic underpinning and distinct evolution of two classes of diterpenoid chemistries within this species.

## Results and discussion

### *T. chamaedrys* genome reveals evidence of tetraploidy

To create a high-quality genome assembly for *T. chamaedrys*, we generated 265 Gbp of long reads using Oxford Nanopore Technology and 95 Gbp of short reads with Illumina sequencing. GenomeScope estimated the *T. chamaedrys* genome size at approximately 1.7 Gbp with low heterozygosity (0.14%; Supplemental Figure 1). Assembly, polishing, and removal of contigs shorter than 10 Kbp resulted in 3162 contigs (Supplemental Table 1) with a final assembly size of 2.9 Gbp (Supplemental Figure 2). Benchmarking Universal Single-Copy Orthologs (BUSCO; Manni et al., 2021) analysis with 2326 total BUSCO genes (eudicots_odb10) revealed 2274 (97.8%) complete orthologs, of which 60 (2.6%) were single copy, 2214 (95.2%) were duplicated, 8 (0.3%) were fragmented, and 44 (1.9%) were missing. Annotation of protein-coding genes identified 128 111 high-confidence genes. BUSCO analysis of the annotation revealed a similar set of statistics, with 2210 (95.0%) complete orthologs, 88 (3.8%) single copies, 2122 (91.2%) duplicated, 21 (0.9%) fragmented, and 95 (4.1%) missing. Overall, this demonstrates a high-quality assembly and annotation of the *T. chamaedrys* genome.

**Figure 2:**
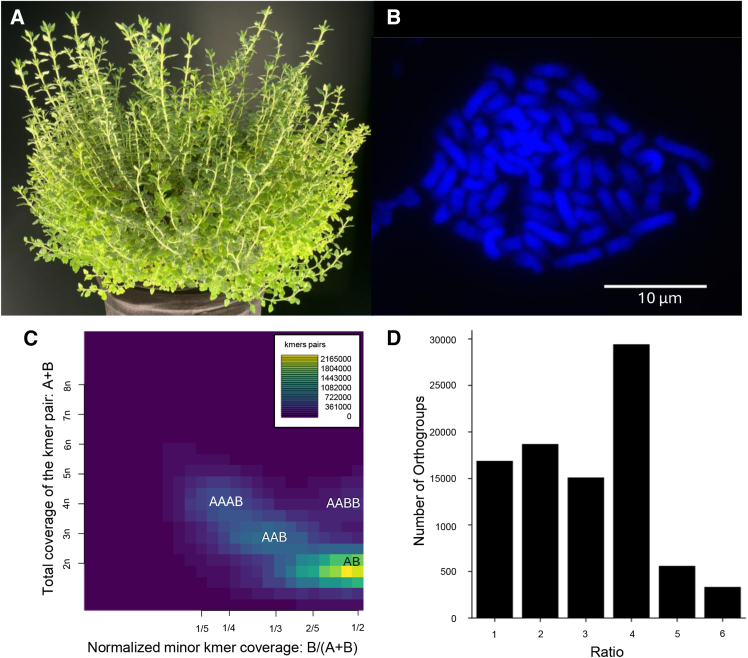
The tetraploid genome of *T. chamaedrys*. **(A)** Image of mature *T. chamaedrys* shrub. **(B)** A representative metaphase cell prepared from a root tip. **(C)** Smudgeplot analysis showing evidence for genome duplication, with k-mers present at 4n configurations AAAB and AABB. **(D)** Orthogroup proportions between *T. chamaedrys* and *A. thaliana*. Approximately 3000 orthogroups have four times as many orthologs in *T. chamaedrys* as in *A. thaliana.*

The presence of a highly duplicated BUSCO score suggests a recent WGD event, which is additionally corroborated by Smudgeplot k-mer analysis of genome duplication (Figure 2C; Ranallo-Benavidez et al., 2020). Polyploids frequently have highly divergent subgenomes, which can lead to an underestimation of shared k-mers (Supplemental Figure 3; Ranallo-Benavidez et al., 2020). The presence of a smudge at the “AAAB” position, coupled with the trace presence of “AABB,” produced a strong signal at 4n coverage, indicating that *T. chamaedrys* is a tetraploid. This is consistent with OrthoFinder (Emms and Kelly, 2019) analysis comparing *T. chamaedrys* to *Arabidopsis thaliana* (*A. thaliana),* which revealed a predominant 4:1 ratio of orthologs, with weaker evidence for 2:1 and 3:1 ratios (Figure 2D). Furthermore, a chromosome count in dividing root tip cells and comparison to the closely related diploid relative *T. marum* (2n = 34; Smit et al., 2024) revealed that the majority of metaphase cells contained 2n = 62 for *T. chamaedrys* (Figure 2B), which is also consistent with a recent WGD event leading to tetraploidy. Previous chromosome counting efforts have shown this species to be variable in chromosome number (2n = 32–96; Ranjbar et al., 2018); the genome k-mer analysis, orthology, and cytogenetic evidence together support tetraploidy.

The evolutionary split between *T. marum* and *T. chamaedrys* is estimated at approximately 4 million years ago (Salmaki et al., 2016), suggesting that the WGD event within the *T. chamaedrys* lineage is relatively recent. Meiotic abnormalities, cell architecture changes, and genetic instability are among the detrimental side effects of WGD (Osborn et al., 2003; De Storme and Mason, 2014; Wang et al., 2021; Blasio et al., 2022), and many polyploids undergo re-diploidization to mitigate these effects (Li et al., 2021; Wang et al., 2021). This recent tetraploid genome may be a fleeting snapshot capturing one of the many polyploidization events that are widespread across plant lineages. Therefore, the data we provide may inform future studies on the effects of polyploidization.

### Phylogenetic evidence shows clustering and expansion of diTPSs in *Teucrium*

**Figure 3:**
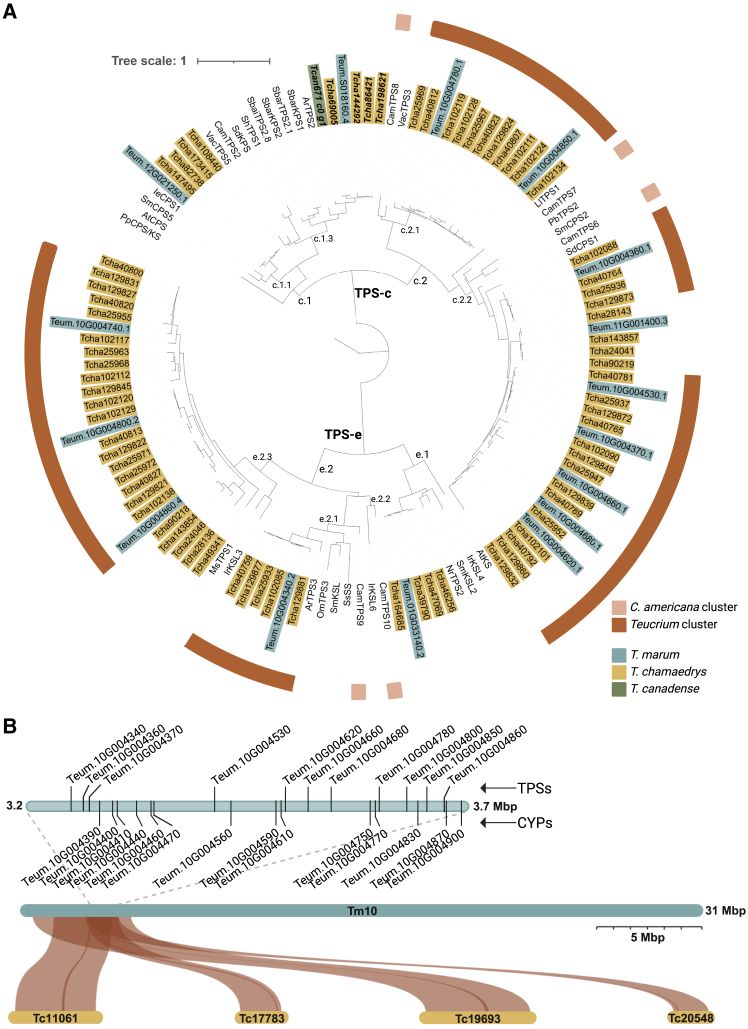
Phylogenetic analysis of the diterpene gene content in three *Teucrium* species. **(A)** This tree is rooted by the class II/class I bifunctional *ent*-kaurene synthase from *Physcometrium patens*. Genes from *T. chamaedrys* are in gold, *T. marum* in blue, and *T. canadense* in green. Those without highlights are previously characterized diTPSs from other Lamiaceae species and *A. thaliana*. Bolded genes were functionally characterized in this study. Red and pink rings denote physical clustering in the genome of *Teucrium* and *C. americana,* respectively. Clades are labeled according to Johnson et al. (2019). Figure was created with iTOL and BioRender.com. **(B)** Syntenic analysis between closely related *T. marum* (blue) and *T. chamaedrys* (gold) show a 1:4 syntenic relationship in a genomic region containing the majority of diTPSs genes. Inset shows the TPSs and CYPs (cytochromes P450) present in the *T. marum* cluster. Designated *T. chamaedrys* nomenclature (3A), Tca40(___), Tca12(___), Tca20(___), and Tca10(___), individually clustered TPS in four loci; (3B) Tc(_____), Tm10 corresponding to individual contigs. Figure was created with SynVisio and BioRender.com.

We estimated the phylogenetic relationships among 90 putative diTPS sequences in three *Teucrium* species (*T. chamaedrys*, *T. marum*, and *T. canadense*) alongside a set of functionally characterized diTPSs from other species in the Lamiaceae family and *A. thaliana* (Supplemental Table 2). One locus in *T. marum* (*Teum.10G004340.2–Teum.10G004860.4*) accounts for 11 of the 15 predicted diTPSs. Similarly, four loci within *T. chamaedrys* (*Tcha40759–Tcha40827*, *Tcha129821–Tcha129881*, *Tcha25933–Tcha25972*, and *Tcha102085–Tcha102138*) account for 53 of the 74 predicted diTPSs, and they cluster across the phylogeny with corresponding orthologs in *T. marum* (Figure 3A).

There is clear synteny between the four genomic regions harboring these diTPSs in *T. chamaedrys* and the corresponding region in *T. marum* (Figure 3B), where *Tcha40759–Tcha40827* are located on contig Tc20548, *Tcha129821–Tcha129881* are on Tc17783, *Tcha25933–Tcha25972* are on Tc11061, and *Tcha102085–Tcha102138* are on Tc19693. This syntenic region contains predicted enzymes that include both class II and class I mechanisms, which is evidence for a large BGC. Interestingly, these clustered genes appear to be part of a Lamiaceae-wide miltiradiene-producing BGC (Bryson et al., 2023), as the clustered *Teucrium* genes are in the same phylogenetic clade (Supplemental Figures 4 and 5). Additionally, this BGC contains around 15 predicted CYPs from the CYP71 clan, which are often involved in diterpenoid metabolism and are also present in the Lamiaceae-wide BGC (Supplemental Figure 6). This *T. marum* cluster appears to form one of the largest diTPS BGCs to date, spanning around 500 Kbp.

Introducing genetic redundancy can lead to diversity in specialized metabolic pathways by relieving selective pressure (Ohno, 1970; Birchler and Yang, 2022). The high number of diTPS sequences in *T. chamaedrys* and *T. marum* reflects a major expansion of specialized metabolism (Figure 3A). *Teucrium* is among the top five Lamiaceae genera in unique diterpene skeleton production (Johnson et al., 2019), and the sheer number of predicted diTPSs in *T. chamaedrys* supports this. Phylogenetic blooms in a species can be attributed to tandem duplication and neofunctionalization, which appears to be the case here, with the large majority of diTPSs appearing to predate the speciation of *T. chamaedrys* and *T. marum* and the WGD event, further increasing the number present in *T. chamaedrys*. The abundance of diTPS sequences in *Teucrium* illustrates the vast diversity of diterpenoids harbored in these species, especially *T. chamaedrys*. The sequences from *T. canadense* were derived from transcriptomic rather than genomic data and therefore may not show a complete picture of diTPS diversity in this species. A higher proportion of diTPSs present in *T. chamaedrys* further suggests WGD, and syntenic analysis corroborates this.

Most plant species have two diTPSs that biosynthesize the initial pathway toward gibberellic acid: one in the phylogenetic clade TPS-e.1 and one in TPS-c.1, corresponding to class I and class II enzymes, respectively. The same 1:4 ratio observed in the BGC is seen in gibberellic acid synthesis genes as well (Figure 3A; TPS-c.1.1 and TPS-e.1). Where one TPS-e.1 is usually seen, there are four in *T. chamaedrys*, and the same is true for TPS-c.1 (Figure 3A). Given the haploid nature of the *T. marum* genome assembly (Smit et al., 2024), this 1:4 ratio is consistent with a WGD event present specifically in the lineage of *T. chamaedrys*.

### Biochemical analysis reveals the basis of clerodane metabolism in *T. chamaedrys*

**Figure 4:**
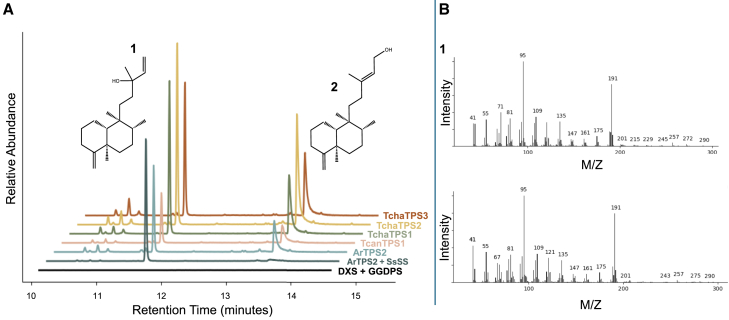
Extracted ion chromatogram (191 *m/z*) demonstrating iso-kolavenyl diphosphate synthase activity. **(A)** Extracted ion chromatograms were stacked and shifted to facilitate comparison of products. Tested enzymes TchaTPS1, TchaTPS2, TchaTPS3, and TcanTPS1 were compared to the known iso-KDP synthase, ArTPS2, and the negative control, DXS+GGDPS. DXS+GGDPS is present in all samples. The peak at ∼11.5 min corresponds to iso-kolavelool (1), and the peak at ∼13.5 min corresponds to iso-kolavenol (2). **(B)** Representative mass spectra of ArTPS2 corresponding to iso-kolavelool (1) and iso-kolavenol (2) peaks. Mass spectra of all relevant peaks are provided in Supplemental Figure 9. A representative chromatogram of three replicates is shown. Compound identification, level 1 (authentic standard, retention time, fragmentation pattern, *m/z*, high-resolution GC–MS data, Supplemental Table 4).

To better understand clerodane representation in *Teucrium*, we investigated the enzyme activity of four predicted clerodane synthase homologs in *T. chamaedrys* and one in *T. canadense*: TchaTPS1, TchaTPS2, TchaTPS3, and TcanTPS1. The fourth predicted clerodane synthase homolog from *T. chamaedrys* was determined to be inactive, with low expression in the plant (Tcha144292; Supplemental Figure 7). To functionally characterize each putative clerodane synthase, we used an *Agrobacterium*-mediated *Nicotiana benthamiana* transformation system in direct comparison with relevant published reference enzymes. We also co-expressed each enzyme with sclareol synthase (SsSS), a promiscuous class I diTPS that, in this context, produces exclusively iso-kolavelool from iso-KDP (Caniard et al., 2012). This allowed us to determine that the product at 11.5 min (1) was iso-kolavelool (neo-cleroda-4(18),14-dien-13-ol), and the other major product at 13.5 min (2) was iso-kolavenol, based on comparison with the reference class II enzyme ArTPS2 (Figure 4A; Supplemental Figure 8; Johnson et al., 2019; mass spectra for 1 and 2 are given in Figure 4B). The mixture of products in runs without SsSS occurs as a result of dephosphorylation catalyzed by non-specific endogenous enzymes in *N. benthamiana* (Supplemental Figure 9). SsSS specifically produces iso-kolavalool as opposed to promiscuous cleavage by the endogenous *N. benthamiana* enzymes. Therefore, all active enzymes were found to produce iso-KDP, and none of the enzymes yielded conclusive evidence of (−)-KDP.

The presence of iso-KDP synthases in *T. chamaedrys* is not surprising, given that an evolutionarily close relative, *A. reptans*, possesses an ortholog, ArTPS2. Additionally, various *Teucrium* furanoclerodanes have been reported with a 4,18 double bond, 4,18 epoxides, and C18 esters that lack a C3–C4 double bond, features that presumably come from an iso-KDP precursor. All reported *T. chamaedrys* clerodanes are heavily modified and lack either the 3,4 or the 4,18 double bond but do have various C18 ester linkages (Dictionary of Natural Products 30.2), suggesting that they are likely formed by an iso-KDP precursor.

While we found no evidence of a dedicated (−)-KDP synthase in *T. chamaedrys*, some Lamiaceae species do contain (−)-KDP synthases and accumulate furanoclerodanes, including *Teucrium* species with a 3,4 double bond (Dictionary of Natural Products 30.2). Given the presence of iso-KDP-derived chemistries in *T. chamaedrys*, either there has been a loss of the (−)-KDP enzyme in certain lineages or specific amino acid substitutions in the enzyme alter the products. Deprotonation of C3, rather than C18, would most likely require a slight positional shift, on the order of a few angstroms, of the base-acting residue to alter which proton is abstracted. It has been shown that blocking the deprotonation site of an *ent*-copalyl diphosphate synthase with a single amino acid substitution can convert the *ent-copalyl diphosphate* synthase into a (−)-KDP synthase (Potter et al., 2016). A third possibility is that another unrelated TPS-c may have convergently evolved (−)-KDP synthase activity.

This study represents the first functional characterization of diTPSs in *Teucrium*, laying the groundwork for future characterization of enzymes involved in subsequent steps of diterpenoid metabolism, such as clerodane-derived compounds like Teucrin, chamaedrosides, and neo-clerodanes. Understanding the natural biosynthetic pathways of these medicinally relevant compounds provides an important first step toward the biotechnological production and utilization of these terpenes in medicine and beyond.

## Methods

### Plant growth conditions, tissue collection, and storage

The *T. chamaedrys* plant was purchased from Mountain Valley Growers (California, USA) and grown in a greenhouse. For DNA extraction, the plant was dark adapted for 72 h prior to harvesting. Healthy, mature leaves were collected, flash frozen in liquid nitrogen, and stored at −80°C. For RNA extraction, healthy, mature leaves and thoroughly rinsed roots were collected, flash frozen in liquid nitrogen, and stored at −80°C.

### Nucleotide isolation

High-molecular-weight genomic DNA was extracted from *T. chamaedrys* leaves using a modified CTAB-based protocol (cetyltrimethylammonium bromide) (Li et al., 2020; Longley et al., 2023). Briefly, frozen tissue was ground into a fine powder with a mortar and pestle in liquid nitrogen and resuspended in a nuclear isolation buffer. After nuclei were isolated, CTAB was added, and high-molecular-weight nucleic acids were extracted with chloroform and isoamyl alcohol, washed with isopropanol, and treated with RNase (Thermo Fisher Scientific, MA, USA). Genomic DNA for short-read sequencing was extracted using the DNeasy Plant Mini Kit (QIAGEN, Hilden, Germany).

### Library preparation and sequencing

DNA libraries for long-read sequencing with Nanopore (Oxford Nanopore Technologies, USA) were prepared using the Oxford Nanopore SQK-LSK114 Ligation Sequencing Kit v.14, and the library was loaded onto a PromethION FLO-PRO114M (R10.4.1) flow cell. Prior to long-read sequencing, the DNA was processed with the Standard Short Read Eliminator Kit (Circulomics, MD, USA). MinKNOW (v.22.10.07) was used for sequencing control, and base calling was performed with Guppy (v.6.3.9) using the high-accuracy model.

For short-read sequencing, library preparation was performed using the Roche Kapa HyperPrep DNA Library Kit with Unique Dual Index adapters (Sigma-Aldrich, MO, USA). The completed library was assessed for quality and quantified using Qubit dsDNA HS, Agilent 4200 TapeStation HS DNA1000, and Invitrogen Collibri Illumina Library Quantification qPCR assays. The sample was loaded onto one lane of an Illumina v.1.5 S4 flow cell using the Xp Workflow. Sequencing was performed in a 2× 150 bp paired-end format using a NovaSeq 6000 (v.1.5) 300 cycle reagent cartridge (Illumina, CA, USA). Base calling was performed using Illumina Real Time Analysis (v.3.4.4), and output from the Real Time Analysis software was demultiplexed and converted to FASTQ format with Illumina’s Bcl2fastq (v.2.20.0).

### Genome size and heterozygosity estimation

Jellyfish (v.2.3.0; Marçais and Kingsford, 2011) was used to estimate genome size and heterozygosity via k-mer analysis. Trimmed and filtered 31-mers from the Illumina DNA libraries were used.

### Ploidy analysis

KMC (Kokot et al., 2017) was also used to count k-mers in the genome using a k-mer length of 31, yielding 4 876 867 453 unique k-mers. K-mer analysis was visualized using GenomeScope (v.1.0; Supplemental Figure 1; Vurture et al., 2017). Subsequently, ploidy was measured using Smudgeplot analysis (Ranallo-Benavidez et al., 2020). The lower coverage threshold cutoff was set to 12, and the upper cutoff was set to 2,800, estimated using “cutoff” from the Smudgeplot suite. The Smudgeplot analysis output was hand-annotated according to Ranallo-Benavidez et al. (2020), as the original output did not include the AAAB annotation (Figure 2; Supplemental Figure 3).

### Genome assembly

Raw Nanopore DNA reads with mean Q-scores greater than 7 were used and processed with Porechop (v.0.2.4) to remove adapters, Chopper (v.0.8.0-0; De Coster et al., 2018) to filter reads shorter than 10 Kb, and Filtlong (v.0.2.0) to remove the worst 10% of reads based on quality. Sequences were then assembled using Flye (v.2.9; Kolmogorov et al., 2019) with a minimum overlap of 5 Kbp, two iterations of polishing, and haplotype retention enabled. The draft assembly was polished once using Medaka (v.1.4.3) and the model “r1041_e82_400bps_hac_g632.” BWA-MEM2 (v.2.0; Vasimuddin and et al., 2019) was used to align the Illumina paired-end reads to the draft assembly for error correction. The resulting draft assembly was polished with one round of Pilon (v.1.24; Walker et al., 2014) using the “diploid” option. Contigs smaller than 100 Kbp were then removed from the assembly. To eliminate potential contamination, Kraken2 (v.2.1.3; Wood et al., 2019) was used with the database “PlusPFP” (https://benlangmead.github.io/aws-indexes/k2). Approximately 0.44% of the assembly was determined to be human and subsequently removed. No further contamination was detected.

### Genome annotation

The draft genome was first mined for *de novo* repeats using Repeat Modeler (v.2.0.2a; Flynn et al., 2020). These *de novo* repeats, along with Viridiplantae repeats from RepBase, were used by Repeat Masker (v.4.1.1; Chen, 2004) to mask the draft genome. Next, RNA sequencing data from *T. chamaedrys* (SRA: PRJNA1124528) mature leaves and roots were aligned to the draft genome using HISAT2 (v.2.1.0; Kim et al., 2019). In addition to this transcript evidence, protein evidence from the closely related species *T. marum* (Smit et al., 2024) was used as an input for BRAKER (v.2.1.6; Altschul et al., 1990; Stanke et al., 2006; 2008; Camacho et al., 2009; Quinlan, 2014; Kovaka et al., 2019; Pertea and Pertea, 2020; Gabriel et al., 2021; Bruna et al., 2023) with the flag “--etpmode" to create initial gene models. These gene models were then fed into MAKER (Law et al., 2015), along with RNA sequencing evidence and protein evidence from *A. thaliana* (TAIR v.11; Cheng et al., 2017) and *T. marum* (Smit et al., 2024), to create a working gene model set (Supplemental Table 3).

This yielded 217 373 working gene models, which were later filtered down to 128 111 high-confidence gene models. Of the original 217 373 gene models, 153 810 had an annotation edit distance score of less than one and/or contained a protein domain, indicating evidence for transcripts or protein homology (Yandell and Ence, 2012). Of those, we kept one gene model per locus, yielding 144 380 gene models. Although a repeat-masked genome was used initially, we found additional transposable element-related genes, which, when removed, yielded 134 486 gene models. All genes shorter than 300 bp were then removed, leaving 128 264 gene models. Finally, we removed non-plant contamination according to Kraken2 (Wood et al., 2019) for a final high-confidence gene model count of 128 111.

### Chromosome counting

Root tips were harvested from greenhouse-grown rooted cuttings and pretreated with nitrous oxide at a pressure of 160 psi (approximately 10.9 atm) for 40 min. Subsequently, the root tips were fixed in a solution of three parts ethanol to one part acetic acid and maintained at 22°C until enzymatic treatment. An enzymatic solution containing 4% cellulase (Yakult Pharmaceutical, Tokyo, Japan), 2% pectinase (Plant Media, Dublin, OH, USA), and 2% pectolyase (Sigma Chemical, St. Louis, MO, USA) was used to digest the root tips for 50 min at 37°C. Chromosomes were prepared using a stirring method as described by Xin et al. (2020) and counterstained with 4′,6-diamidino-2-phenylindole in VectaShield antifade solution (Vector Laboratories, Burlingame, CA, USA). Images were captured with a QImaging Retiga EXi Fast 1394 CCD camera (Teledyne Photometrics, Tucson, AZ, USA) attached to an Olympus BX51 epifluorescence microscope. Image processing was performed using Meta Imaging Series 7.5 software, and the final image contrast was adjusted using Adobe Photoshop (Adobe, San Jose, CA, USA). Chromosome counting was conducted on at least 10 metaphase spreads.

### Phylogeny

*T. canadense* reads were downloaded from the NCBI Sequence Read Archive (SRA) database (SRR5150734), and the split read files were assembled into a *de novo* transcriptome using Trinity (v.2.9.1; Grabherr et al., 2011). The resulting mRNA was filtered for the longest open reading frame and translated into protein sequences using TransDecoder (v.2.1.0; Haas et al., 2013; https://github.com/TransDecoder/TransDecoder). *T. marum* gene models were downloaded from Figshare (https://figshare.com/articles/dataset/Teucrium_marum_genome_assembly/25109411). The representative gene models from each of the three *Teucrium* species were queried using BLAST (BLAST+ v.2.13.0, e value = 1e−20; Camacho et al., 2009) against a bait set of 34 functionally characterized diTPSs (Supplemental Table 2). Resulting protein matches were identified and combined with the bait set. Multiple sequence alignments were generated using ClustalOmega (v.1.2.4; Sievers et al., 2011), and phylogenetic trees were generated using RAxML using the model “protgammaauto,” algorithm “a,” and 100 bootstrap replicates (v.8.2.12; Stamatakis, 2014).

### Synteny

The BLAST function makeblastdb (e value = 1e−10, 5 alignments) was used to create protein databases for *T. chamaedrys* and *T. marum* (Smit et al., 2024). Syntenic analysis was performed using the standard MCScanX pipeline (match score = 50; match size = 5; gap penalty = −1; overlap window = 5; e value = 1e−5; max gaps = 25; Wang et al., 2012). Results were visualized using SynVisio (Bandi and Gutwin, 2020).

### Cloning and transient expression

Candidate enzymes from *T. chamaedrys* were synthesized (Twist Bioscience, CA, USA) and cloned into the plant expression vector pEAQ-HT (Sainsbury et al., 2009) for use in transient expression in *N. benthamiana*. Sequences were validated via Sanger sequencing. *N. benthamiana* plants were grown for 4–5 weeks in a controlled growth room under a 12-h light and 12-h dark (22°C) cycle before infiltration. Coexpression constructs were transformed separately into *Agrobacterium tumefaciens* strain LBA4404. Cultures were grown overnight at 30°C in lysogeny broth containing 50 μg/ml kanamycin and 50 μg/ml rifampicin. Cultures were collected by centrifugation and washed twice with approximately 10 ml water before being resuspended and diluted to an OD600 of 1.0 in water with 200 μM acetosyringone. Cultures were incubated at 30°C for 1–2 h, after which equal volumes of each culture were mixed for each combination of enzymes. *N. benthamiana* leaves were infiltrated on the underside (abaxial side) with a 1-ml syringe. All gene constructs were co-infiltrated with two genes encoding rate-limiting steps in the upstream 2-C-methyl-D-erythritol 4-phosphate pathway, *Plectranthus barbatus* 1-deoxy-D-xylulose-5-phosphate synthase and GGDP synthase, to boost production of the diterpene precursor GGDP (Andersen-Ranberg et al., 2016). Plants were returned to the controlled growth room for 5 days. Approximately 200 mg of fresh weight from three separate infiltrated leaves was extracted with 1.5 ml hexane overnight at room temperature. Plant material was collected by centrifugation, and the organic phase was removed for gas chromatography–mass spectrometry (GC–MS) analysis.

### GC–MS analysis

All GC–MS analyses were performed on an Agilent 7890 A GC with an Agilent VF-5ms column (30 m × 250 μm × 0.25 μm, with 10 m EZ-Guard) and an Agilent 5975 C detector. The inlet was set to 250°C with splitless injection of 1 μl using He carrier gas (flow rate = 1 ml/min). The detector was activated following a 4-min solvent delay. All assays and tissue analyses used the following method: temperature ramp start 40°C, hold 1 min, 40°C/min to 200°C, hold 4.5 min, 20°C/min to 240°C, 10°C/min to 280°C, 40°C/min to 320°C, and hold 5 min. The MS scan range was set to 40–400.

## Data and code availability

The data supporting the findings of this work are available within the paper and supplemental information. Raw genomic WGS and FLcDNA (full-length cDNA) reads generated in this study have been deposited in the NCBI SRA under accession number SRA: PRJNA1246154. Sequences for the four functionally characterized enzymes are available in the NCBI BankIt under accession numbers PQ246887–PQ246890. The Genome assembly, annotation, raw GC–MS, and a list of *Teucrium* sequences used in Figure 3 are available through our Dryad Repository (https://doi.org/10.5061/dryad.4mw6m90kp). A voucher specimen of *T. chamaedrys* has been deposited at the Michigan State University Herbarium and can be found under catalog number MSC0291921 and secondary catalog number 415574.

## Funding

A.E.B. and N.S. would like to acknowledge the generous support of the Neogen Land Grant Prize, an endowed grant program administered by the Office of Research and Innovation at Michigan State University (MSU), which supports graduate students in translating their research into real-world applications that positively impact society and the US economy. A.E.B. and D.M. are funded by a National Science Foundation (NSF)-IMPACTS Training Grant (DGE-1828149). A.E.B., D.M., and B.H. are funded by NSF Dimensions of Biodiversity (DEB 1737898). N.S. is supported by the 10.13039/100000057National Institute of General Medical Sciences of the 10.13039/100000002National Institutes of Health under award number T32 GM110523*.* B.H. and N.S. gratefully acknowledge the 10.13039/100000015US Department of Energy Great Lakes Bioenergy Research Center Cooperative Agreement DE-SC0018409. B.H. also acknowledges startup funding from the Department of Biochemistry and Molecular Biology at MSU and support from 10.13039/100011138AgBioResearch (MICL02454), as well as a generous endowment from James K. Billman, Jr., MD. B.H. is also supported in part by the 10.13039/100000001National Science Foundation under Grant number 1737898. C.R.B. acknowledges funding from the 10.13039/100007699University of Georgia, the Georgia Research Alliance, and Georgia Seed Development. J.J. acknowledges support from the NSF under grant number ISO-2029959.

## Acknowledgments

We would like to thank Matt Chansler, Jennifer S. Apland, and Alan Prather for processing the herbarium specimen; Emily R. Lanier for RNA extraction; Britta Hamberger for plant care; and Patrick Edger and Jim Leebens-Mack for discussions. This work was supported in part by computational resources and services provided by the Institute for Cyber-Enabled Research at MSU, and by resources and technical expertise from the Georgia Advanced Computing Resource Center, a partnership between the Office of the Vice President for Research and the Office of the Vice President for Information Technology at the University of Georgia. We thank the MSU RTSF Genomics Core for sequencing services and the MSU Mass Spectrometry and Metabolomics Core Facility for access to the GC–MS instrumentation. MSU occupies the ancestral, traditional, and contemporary lands of the Anishinaabeg–Three Fires Confederacy of Ojibwe, Odawa, and Potawatomi peoples. MSU resides on land ceded in the 1819 Treaty of Saginaw. Any opinions, findings, and conclusions or recommendations expressed in this material are those of the authors and do not necessarily reflect the views of the National Science Foundation. The content is solely the responsibility of the authors and does not necessarily represent the official views of the National Institutes of Health. No conflict of interest declared.

## Author contributions

Conceptualization, A.E.B., N.S., and B.H.; investigation and methodology, A.E.B., D.M., K.L.C., and J.P.H.; genomic analysis, A.E.B. and D.M.; phylogenetic analysis and biochemical assays, A.E.B.; chromosome squash, H.X.; writing – original draft, all authors; writing – review & editing, A.E.B. and B.H.; funding acquisition, A.E.B., N.S., and B.H.; resources, B.H., J.J., and C.R.B.; supervision, J.J., C.R.B., and B.H.

## Supplemental information

Document S1. Supplemental Figures 1–9 and Supplemental Tables 1–4

Document S2. Article plus supplemental information
