# Supplementary material for: A high-quality genome assembly of the tetraploid Teucrium chamaedrys unveils a recent whole-genome duplication and a large biosynthetic gene cluster for diterpenoid metabolism
Source: Plant Commun. 2025 Jun 3;6(8):101393. doi: 10.1016/j.xplc.2025.101393 (PMC12365839; doi:10.1016/j.xplc.2025.101393)
Supplement: Document S1. Supplemental Figures 1–9 and Supplemental Tables 1–4 [file mmc1.pdf]

Plant Communications, Volume *6*

Supplemental information

A high-quality genome assembly of the tetraploid *Teucrium chamaedrys* unveils a recent whole-genome duplication and a large biosynthetic gene cluster for diterpenoid metabolism

Abigail E. Bryson, Kevin L. Childs, Nicholas Schlecht, Davis Mathieu, John P. Hamilton, Haoyang Xin, Jiming Jiang, C. Robin Buell, and Björn Hamberger

Supplemental Files

A high-quality genome assembly of the tetraploid *Teucrium chamaedrys* unveils a recent whole genome duplication and a large biosynthetic gene cluster for diterpenoid metabolism

Bryson *et al* 2025.

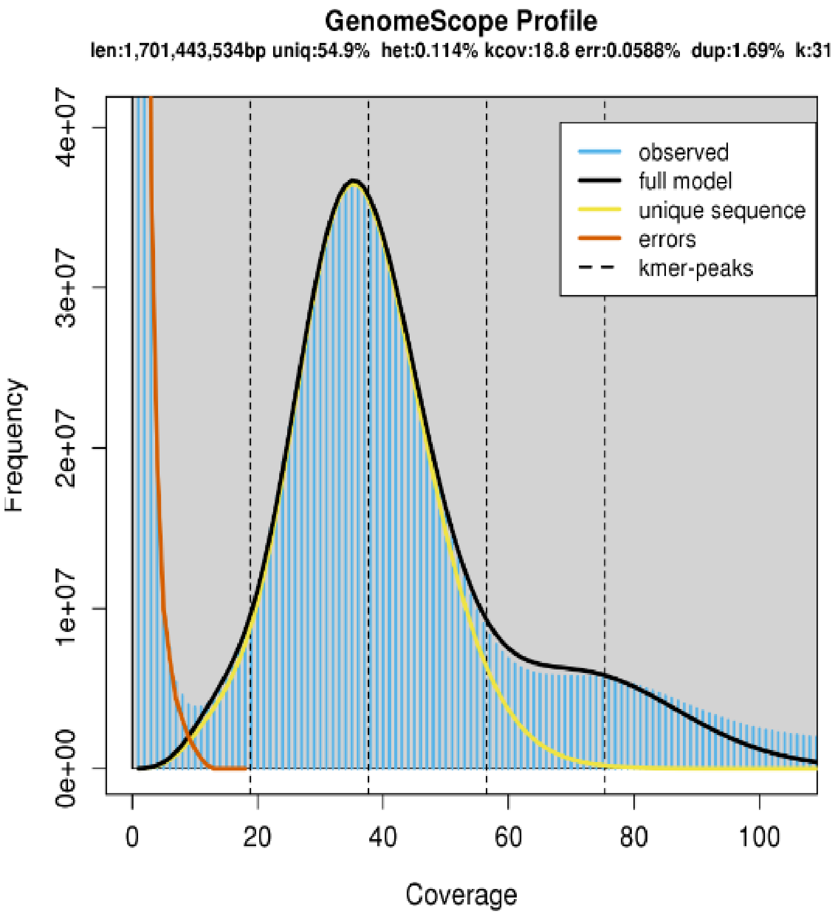

GenomeScope version 1.0
k = 31

property	min	max
Heterozygosity	0.0889729%	0.138491%
Genome Haploid Length	1,680,787,676 bp	1,701,443,534 bp
Genome Repeat Length	758,478,916 bp	767,800,161 bp
Genome Unique Length	922,308,759 bp	933,643,373 bp
Model Fit	90.0569%	97.8819%
Read Error Rate	0.0587909%	0.0587909%

Supplemental Figure 1. GenomeScope results. GenomeScope measures heterozygosity and predicts genome size based on short reads.

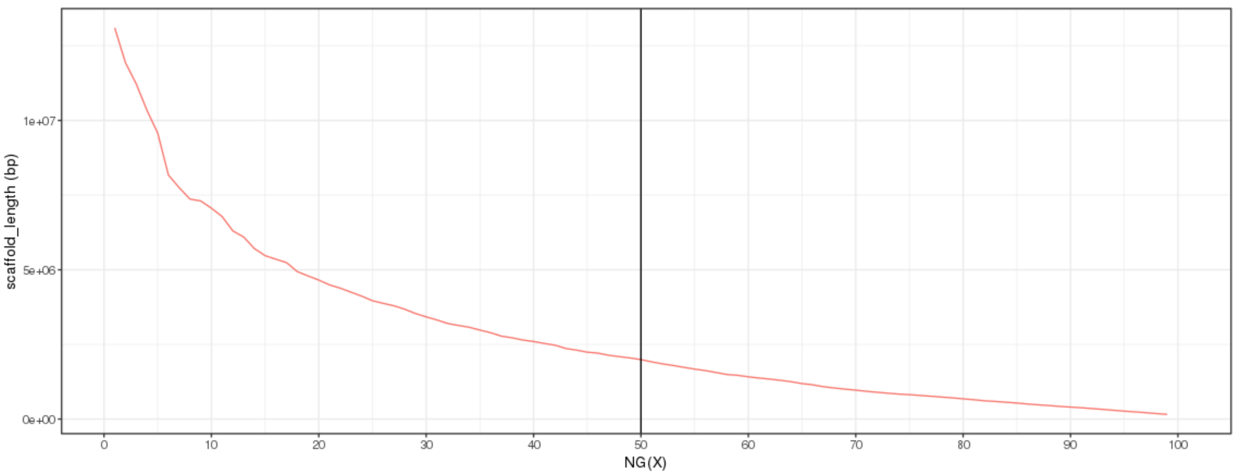

Supplemental Figure 2. Comparison of the genome assembly quality based on NG(X) values. Line represents contig lengths at different NG levels.

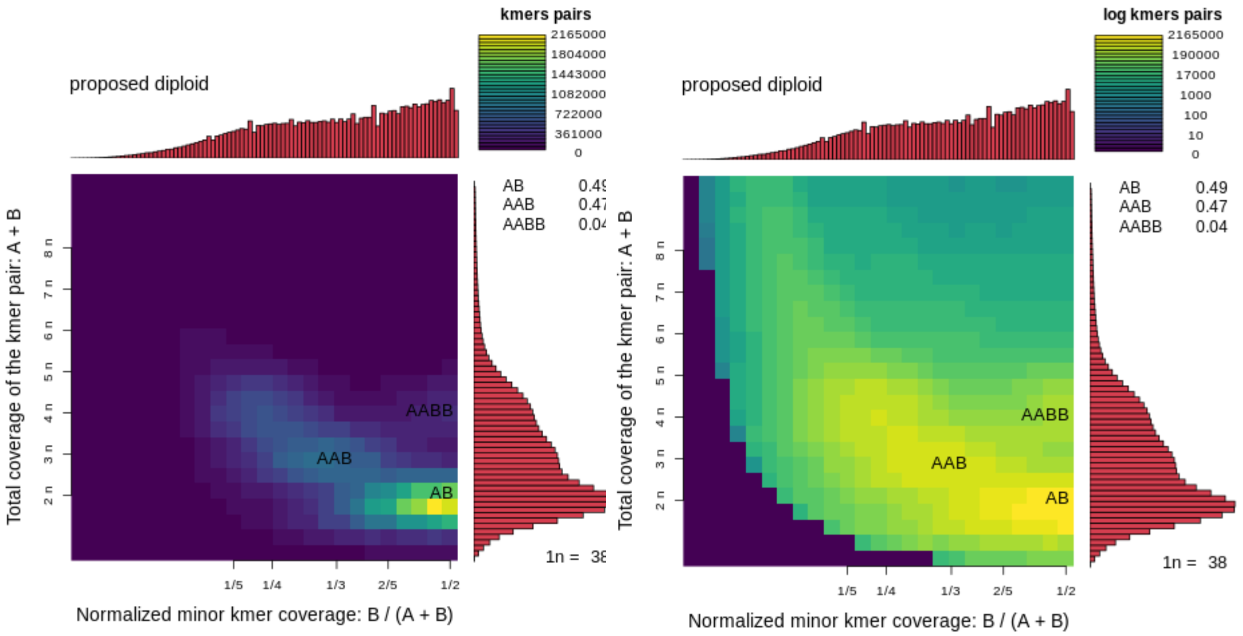

Supplemental Figure 3. Complete and unedited Smudgeplot results. Although this result proposed diploidy, evidence within this figure and across other analyses disagree. Since polyploids can have diverse genomes, sometimes k-mer based analysis can predict diploid for poplyploid species. An unmarked smudge at the 'AAAB' position coupled with the marked smudge at 'AABB' show evidence for 4n coverage of k-mers.

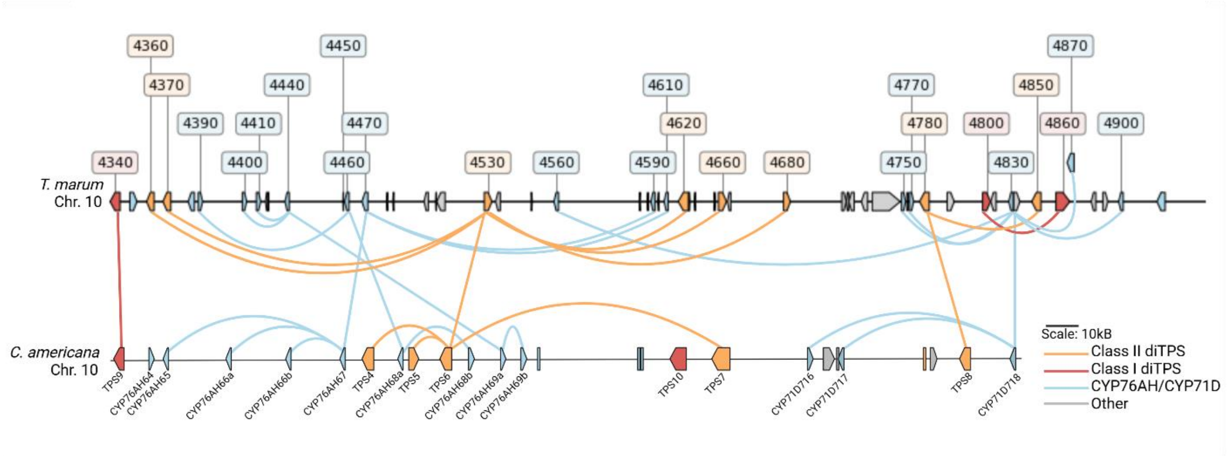

Supplemental Figure 4. Synteny between the BGC regions investigated in *T. marum* compared to *Callicarpa americana*. The top line shows the physical map of the region on chromosome 10 of *T. marum* investigated as a BGC in this study. The bottom line shows the syntenically equivalent region on chromosome 10 in *C. americana*. Synteny lines represent phylogenetic clades and can be referenced in Supplemental Figure 5.

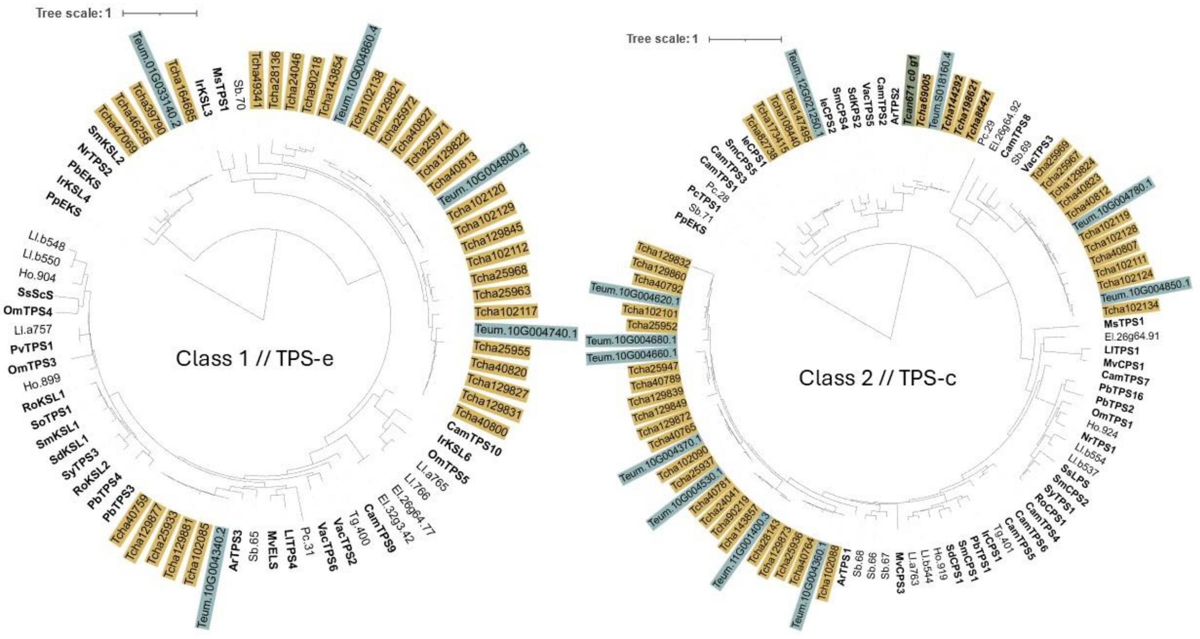

Supplemental Figure 5. Phylogenetics shows relationships of *Teucrium* diTPSs to other mint diTPSs present in a BGC. All *T. marum* (blue) and *T. chamaedrys* (gold) sequences with reference enzymes bolded in addition to sequences found to be part of the Lamiaceae-wide miltiradiene cluster reported in Bryson *et al* 2023. 100 BS confidence.

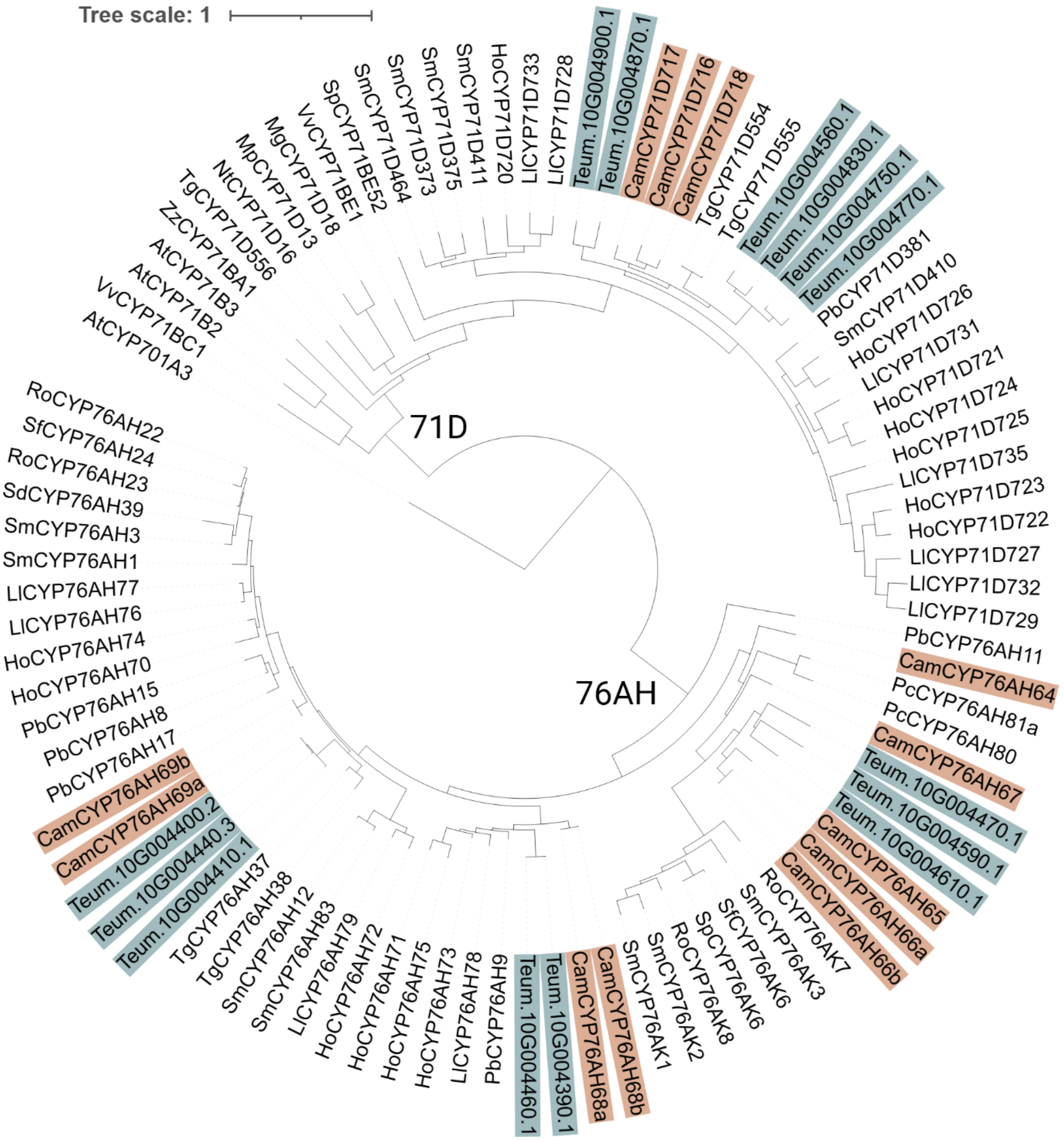

Supplemental Figure 6. Phylogenetics shows relationships of *Teucrium* CYPs to other mint CYPs present in a BGC. *T. marum* (blue) and *C. americana* (rose) sequences in addition to sequences found to be part of the Lamiaceae-wide miltiradiene cluster reported in Bryson *et al* 2023. 100 BS confidence.

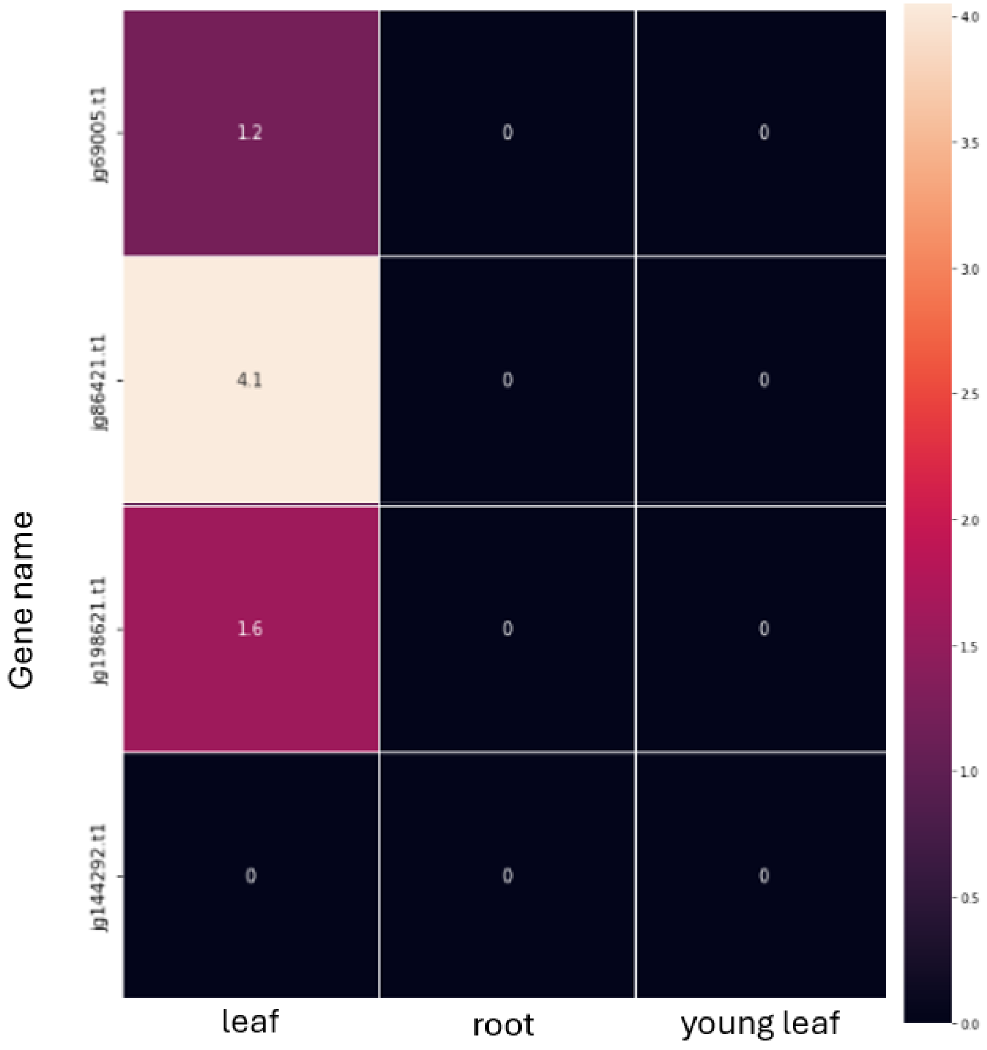

Supplemental Figure 7. RNA expression in *T. chamaedrys* tissues for putative clerodane synthases in this study. Genes characterized in this study only have expression in mature leaves.

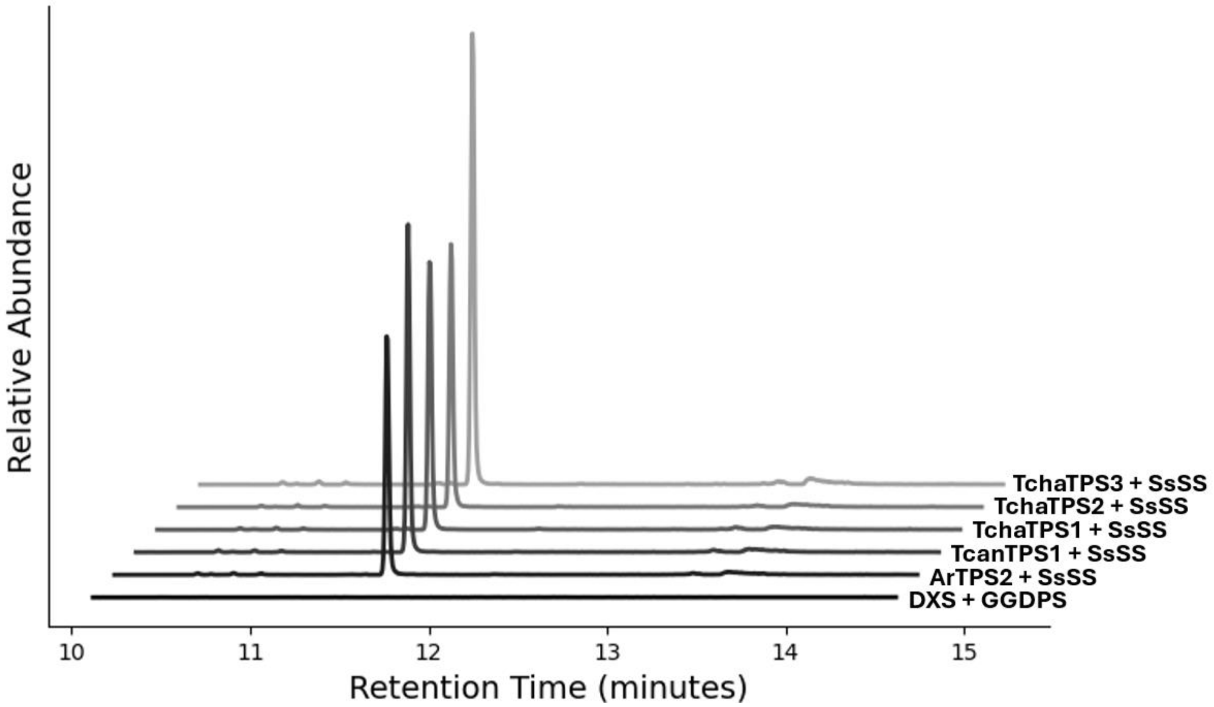

Supplemental Figure 8. Extracted ion chromatogram (191 m/z) demonstrating iso-kolavalool activity. Each EIC was stacked and shifted to compare their products. Peak indicates production of the KDP-derivative, iso-kolavalool. All samples have DXS+GGDPS even if not explicitly stated. Representative chromatograms of at least 3 replicates shown.

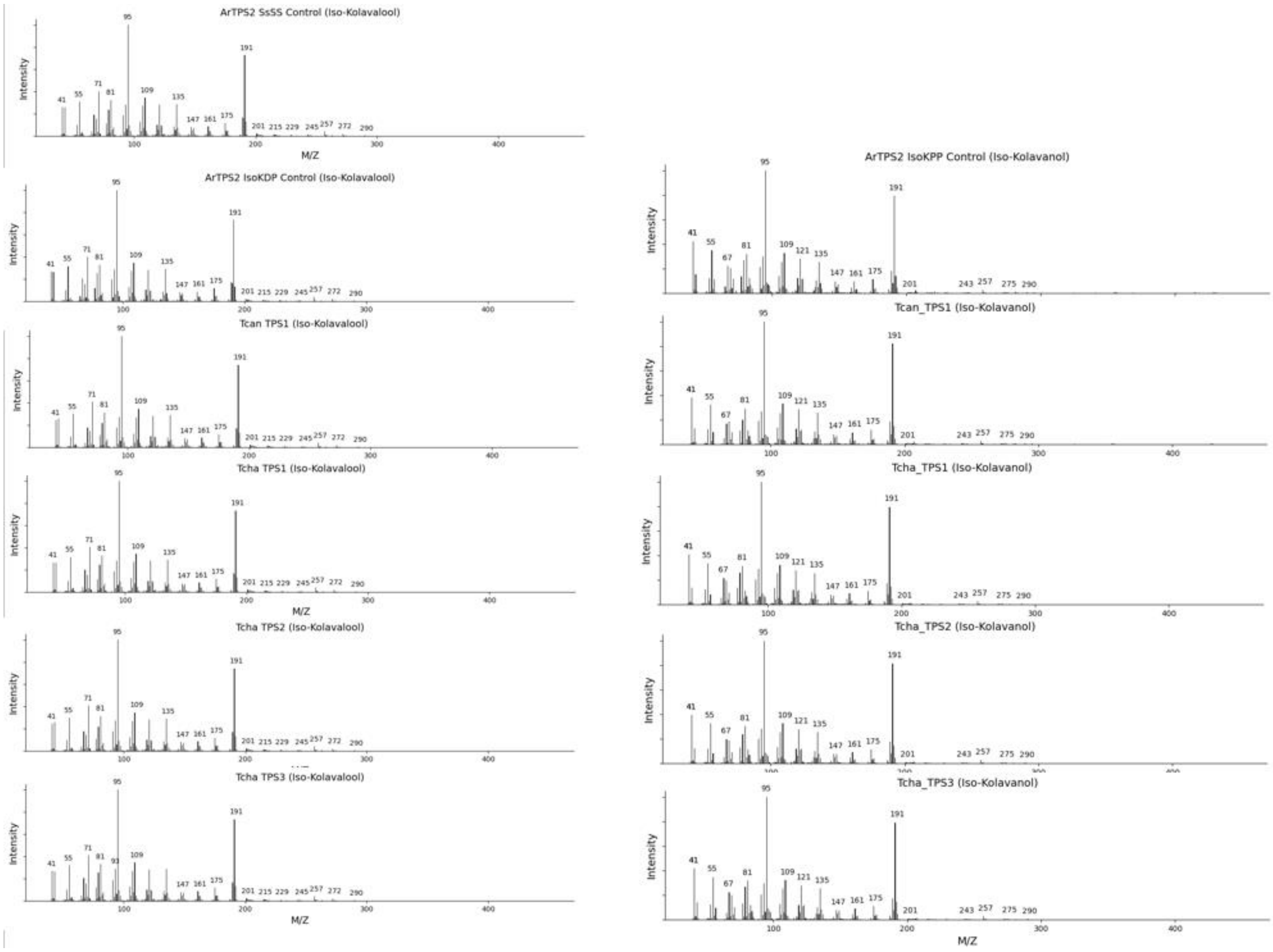

Supplemental Figure 9. Mass spectra for each peak shown in Figure 4. Left column corresponds to iso-kolavalool and the right column corresponds to iso-kolavanol. Both are KDP-derived products.

Total size (bp)	2,926,520,217
Number of contigs	3,148
Largest contig (bp)	16,924,500
N50	1,961,342
N90	385,309
L50	390
L90	1,777
GC Content	39.36%

Supplemental Table 1. Assembly statistics for the genome assembly of *Teucrium chamaedrys*.

Enzyme Name	Species	NCBI GenBank Number
PpCPS/KS	*Physcometrium patens*	BAF61135.1
AtCPS	*Arabidopsis thaliana*	Q38802.1
SmCPS5	*Salvia miltiorrhiza*	AEZ55692.1
IeCPS1	*Isodon eriocalyx*	G3E4M6.1
CamTPS2	*Callicarpa americana*	QMW69082.1
VacTPS5	*Vitex agnus-castus*	A0A2K9RG07.1
SdKPS	*Salvia divinorum*	A0A1S5RW73.1
ShTPS1	*Salvia hispanica*	XP_047942076.1
SbaiTPS2.8	*Scutellaria baicalensis*	UNZ93479.1
SbarKSP2	*Scutellaria barbata*	WJZ49120.1
SbarTPS2.1	*Scutellaria barbata*	UNZ11786.1
SbarKPS1	*Scutellaria barbata*	WJZ49119.1
ArTPS2	*Ajuga reptans*	AZB50378.1
VacTPS3	*Vitex agnus-castus*	A0A2K9RFZ8.1
LlTPS1	*Leonotis Leonurus*	AZB50381.1
CamTPS7	*Callicarpa americana*	UXG91343.1
CamTPS6	*Callicarpa americana*	QMW69083.1
PbTPS2	*Plectranthus barbatus*	AHW04047.1
SmCPS2	*Salvia miltiorrhiza*	AEZ55684.1
SdCPS1	*Salvia divinorum*	APH81399.1
AtKS	*Arabidopsis thaliana*	Q9SAK2.1
IrKSL4	*Isodon rubescens*	A0A1Z3GBK8.1
SmKSL2	*Salvia miltiorrhiza*	H6VLG5.2
NrTPS2	*Nepta racemosa*	AZB50370.1
CamTPS10	*Callicarpa americana*	UXG91345.1
IrKSL6	*Isodon rubescens*	A0A1Z3GCD1.1
CamTPS9	*Callicarpa americana*	UXG91344.1
SsSS	*Salvia sclerea*	G8GJ94.1
OmTPS3	*Origanum majorana*	AZB50371.1
SmSKL	*Salvia miltiorrhiza*	C8XPS0.1
ArTPS3	*Ajuga reptans*	AZB50367.1
IrKSL3	*Isodon rubescens*	A0A1X9ISH5.2
MsTPS1	*Mentha spicata*	AZB50369.1

Supplemental Table 2. Enzyme names, corresponding species, and accession number for all reference sequences used in generating the phylogeny in Figure 3.

Number of gene models	128,111
Average gene length (bp)	2,937.5
Average exons per gene model	4.4
Average exon length (bp)	223.8

Supplemental Table 3. Assembly statistics for the genome annotation of *Teucrium chamaedrys*.

Supplemental Table 4. High resolution mass spectrometry, GC-HRT+ GC/Time-of-Flight MS, given mass defect between predicted ions and measured mass. Details for methods, spectra and data are given in 'Data Availability'.

Sample	masslynx selected region for Calculated mass	masslynx subtracted region for Calculated mass	Predicted Structure	Ion Description	Predicted Monoisotopic mass	Measured mass	Δppm
DXS-GGPPS + ArTPS2	5524-5579	5354-5508 & 5625-5786	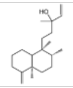	Parent ion	290.261	Below annotaiton threshold	NA
DXS-GGPPS + ArTPS2	5524-5579	5354-5508 & 5625-5786	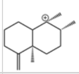	Second largest fragment (decalin core)	191.1794	191.1793	0.52
DXS-GGPPS + ArTPS2	5524-5579	5354-5508 & 5625-5786	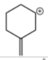	Largest fragment	95.0856	95.0854	2.10
DXS-GGPPS + ArTPS2 + SsSS	5524-5579	5354-5508 & 5625-5786	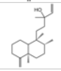	Parent ion	290.261	290.2600	3.45
DXS-GGPPS + ArTPS2 + SsSS	5524-5579	5354-5508 & 5625-5786	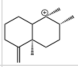	Second largest fragment (decalin core)	191.1794	191.1793	0.52
DXS-GGPPS + ArTPS2 + SsSS	5524-5579	5354-5508 & 5625-5786	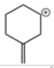	Largest fragment	95.0856	95.0854	2.10
DXS-GGPPS + TchaTPS1	5524-5579	5354-5508 & 5625-5786	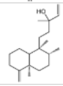	Parent ion	290.261	290.2606	1.38
DXS-GGPPS + TchaTPS1	5524-5579	5354-5508 & 5625-5786	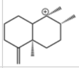	Second largest fragment (decalin core)	191.1794	191.1792	1.05
DXS-GGPPS + TchaTPS1	5524-5579	5354-5508 & 5625-5786	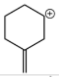	Largest fragment	95.0856	95.0854	2.10
DXS-GGPPS + TchaTPS2	5524-5579	5354-5508 & 5625-5786	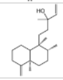	Parent ion	290.261	290.2596	4.82
DXS-GGPPS + TchaTPS2	5524-5579	5354-5508 & 5625-5786	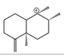	Second largest fragment (decalin core)	191.1794	191.1792	1.05
DXS-GGPPS + TchaTPS2	5524-5579	5354-5508 & 5625-5786	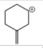	Largest fragment	95.0856	95.0854	2.10
DXS-GGPPS + TchaTPS3	5524-5579	5354-5508 & 5625-5786	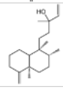	Parent ion	290.261	290.2587	7.92
DXS-GGPPS + TchaTPS3	5524-5579	5354-5508 & 5625-5786	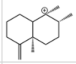	Second largest fragment (decalin core)	191.1794	191.1792	1.05
DXS-GGPPS + TchaTPS3	5524-5579	5354-5508 & 5625-5786	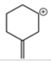	Largest fragment	95.0856	95.0854	2.10
DXS-GGPPS + TcTPS1	5524-5579	5354-5508 & 5625-5786	Picture	Parent ion	290.261	Below annotaiton threshold	NA
DXS-GGPPS + TcTPS1	5524-5579	5354-5508 & 5625-5786	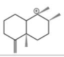	Second largest fragment (decalin core)	191.1794	191.1794	0.00
DXS-GGPPS + TcTPS1	5524-5579	5354-5508 & 5625-5786	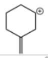	Largest fragment	95.0856	95.0855	1.05
DXS-GGPPS + ArTPS2	6830-6940	6496-6667 & 7015-7230	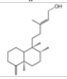	Parent ion	290.261	Below annotaiton threshold	NA
DXS-GGPPS + ArTPS2	6830-6940	5354-5508 & 5625-5787	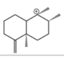	Second largest fragment (decalin core)	191.1794	191.1793	0.52
DXS-GGPPS + ArTPS2 + SsSS	6830-6940	5354-5508 & 5625-5789	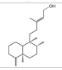	Parent ion	290.261	Below annotaiton threshold	NA
DXS-GGPPS + ArTPS2 + SsSS	6830-6940	5354-5508 & 5625-5790	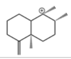	Second largest fragment (decalin core)	191.1794	191.1793	0.52
DXS-GGPPS + ArTPS2 + SsSS	6830-6940	5354-5508 & 5625-5791	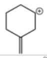	Largest fragment	95.0856	95.0855	1.05
DXS-GGPPS + TchaTPS1	6830-6940	5354-5508 & 5625-5792	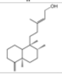	Parent ion	290.261	Below annotaiton threshold	NA
DXS-GGPPS + TchaTPS1	6830-6940	5354-5508 & 5625-5793	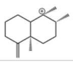	Second largest fragment (decalin core)	191.1793	191.1792	0.52
DXS-GGPPS + TchaTPS1	6830-6940	5354-5508 & 5625-5794	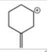	Largest fragment	95.0855	95.0854	1.05
DXS-GGPPS + TchaTPS2	6830-6940	5354-5508 & 5625-5792	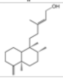	Parent ion	290.261	Below annotaiton threshold	NA
DXS-GGPPS + TchaTPS2	6830-6940	5354-5508 & 5625-5793	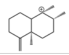	Second largest fragment (decalin core)	191.1794	191.1792	1.05
DXS-GGPPS + TchaTPS2	6830-6940	5354-5508 & 5625-5794	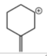	Largest fragment	95.0856	95.0854	2.10
DXS-GGPPS + TchaTPS3	6830-6940	5354-5508 & 5625-5792	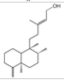	Parent ion	290.261	Below annotaiton threshold	NA
DXS-GGPPS + TchaTPS3	6830-6940	5354-5508 & 5625-5793	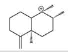	Second largest fragment (decalin core)	191.1794	191.1792	1.05
DXS-GGPPS + TchaTPS3	6830-6940	5354-5508 & 5625-5794	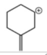	Largest fragment	95.0856	95.0854	2.10
DXS-GGPPS + TcTPS1	6830-6940	5354-5508 & 5625-5792	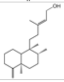	Parent ion	290.261	Below annotaiton threshold	NA
DXS-GGPPS + TcTPS1	6830-6940	5354-5508 & 5625-5793	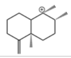	Second largest fragment (decalin core)	191.1794	191.1794	0.00
DXS-GGPPS + TcTPS1	6830-6940	5354-5508 & 5625-5794	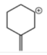	Largest fragment	95.0856	95.0855	1.05

continued from bottom.
