# Supplementary material for: A high-quality genome assembly of the tetraploid Teucrium chamaedrys unveils a recent whole-genome duplication and a large biosynthetic gene cluster for diterpenoid metabolism
Source: Plant Commun. 2025 Jun 3;6(8):101393. doi: 10.1016/j.xplc.2025.101393 (PMC12365839; doi:10.1016/j.xplc.2025.101393)
Supplement: Document S2. Article plus supplemental information [file mmc2.pdf]

Plant Communications
Research article

CellPress
Partner Journal

A high-quality genome assembly of the tetraploid *Teucrium chamaedrys* unveils a recent whole-genome duplication and a large biosynthetic gene cluster for diterpenoid metabolism

Abigail E. Bryson[1], Kevin L. Childs[2], Nicholas Schlecht[1], Davis Mathieu[1], John P. Hamilton[4,5], Haoyang Xin[2], Jiming Jiang[2,3], C. Robin Buell[4,5,6,7] and Björn Hamberger[1,*]

[1]Department of Biochemistry, Michigan State University, East Lansing, MI 48823, USA
[2]Department of Plant Biology, Michigan State University, East Lansing, MI 48823, USA
[3]Department of Horticulture, Michigan State University, East Lansing, MI 48823, USA
[4]Center for Applied Genetic Technology, University of Georgia, Athens, GA 30602, USA
[5]Department of Crop & Soil Sciences, University of Georgia, Athens, GA 30602, USA
[6]Institute of Plant Breeding, Genetics, & Genomics, University of Georgia, Athens, GA 30602, USA
[7]The Plant Center, University of Georgia, Athens, GA 30602, USA
***Correspondence: Björn Hamberger (hamberge@msu.edu)**
https://doi.org/10.1016/j.xplc.2025.101393

ABSTRACT

***Teucrium chamaedrys*, commonly known as wall germander, is a small woody shrub native to the Mediterranean region. Its name is derived from the Greek words meaning "ground oak," as its tiny leaves resemble those of an oak tree. *Teucrium* species are prolific producers of diterpenes, endowing them with valuable properties widely utilized in traditional and modern medicine. Sequencing and assembly of the 3-Gbp tetraploid *T. chamaedrys* genome revealed 74 diterpene synthase genes, with a substantial number of these genes clustered at four synteny genomic loci, each harboring a copy of a large diterpene biosynthetic gene cluster. Comparative genomics revealed that this cluster is conserved in the closely related species *Teucrium marum*. Along with the presence of several cytochrome p450 sequences, this region is among the largest biosynthetic gene clusters identified. *Teucrium* is well known for accumulating clerodane-type diterpenoids, which are produced from a kolavenyl diphosphate precursor. To elucidate the complex biosynthetic pathways of these medicinal compounds, we identified and functionally characterized several kolavenyl diphosphate synthases from *T. chamaedrys*. The remarkable chemical diversity and tetraploid nature of *T. chamaedrys* make it a valuable model for studying genomic evolution and adaptation in plants.**

Key words: Lamiaceae (mint), *Teucrium*, diterpenoid, biosynthetic gene cluster, BGC

Bryson A.E., Childs K.L., Schlecht N., Mathieu D., Hamilton J.P., Xin H., Jiang J., Buell C.R., and Hamberger B. (2025). A high-quality genome assembly of the tetraploid *Teucrium chamaedrys* unveils a recent whole-genome duplication and a large biosynthetic gene cluster for diterpenoid metabolism. Plant Comm. **6**, 101393.

INTRODUCTION

The Lamiaceae (mint) family includes culturally and economically important plants such as peppermint, lavender, sage, rosemary, and teak. It is the third-largest family of flowering plants, with an estimated 7000 species. However, representative genomes are limited, with only about 0.66% (46) published to date. Sampling understudied clades in the Lamiaceae can help elucidate the basis of specialized metabolism, as this family is known to produce nearly 7500 unique plant natural products relevant to human health and industry (Dictionary of Natural Products 30.2). The subfamily Ajugoideae (syn. Teucrioideae) is one such understudied clade, which includes approximately 770 species and only three published genomes (Ritz et al., 2023; Smit et al., 2024). Within Ajugoideae, the polyphyletic *Teucrium* is one of the largest genera, with approximately 300 species. *Teucrium* has been used for millennia, with historical applications such as treating asthma in ancient Greece (Menichini et al., 2009).

 © 2025 The Author(s). Published by Elsevier Inc. on behalf of CAS Center for Excellence in Molecular Plant Sciences, Chinese Academy of Sciences, and Chinese Society for Plant Biology.
This is an open access article under the CC BY license (http://creativecommons.org/licenses/by/4.0/).

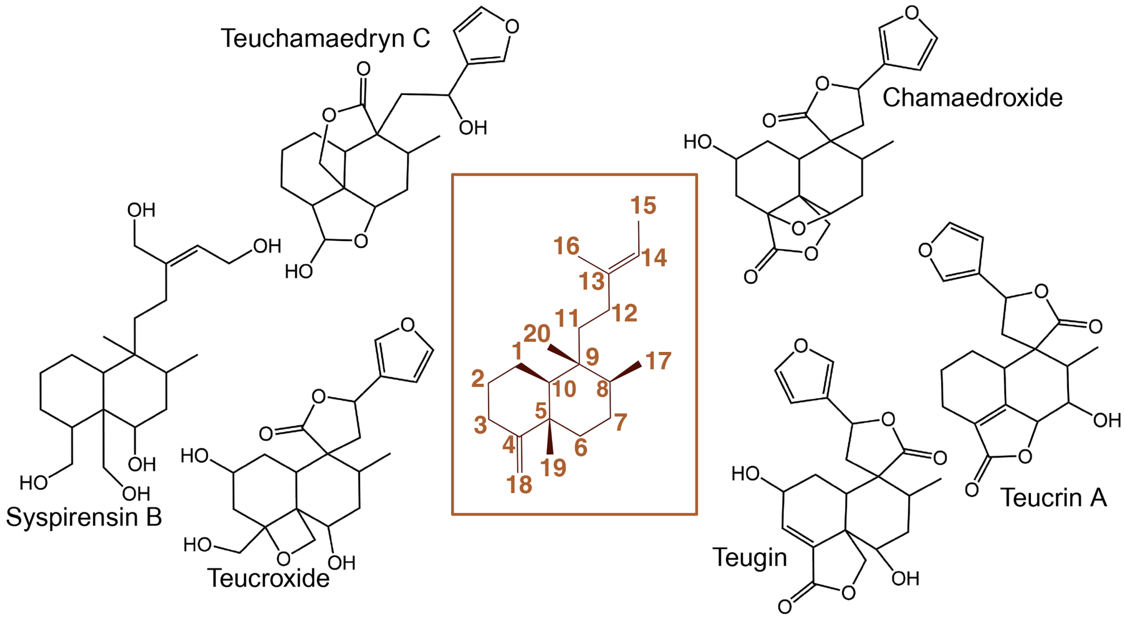

Figure 1. Clerodane skeleton and select clerodanes from *T. chamaedrys*.
Teucrium, specifically *T. chamaedrys*, is rich in clerodane-type diterpenoids. Middle box features numbered carbons on a typical clerodane skeleton.

Teucrium species are also well known for their insect antifeedant activity; allelopathic inhibition of cosmopolitan weeds; antimicrobial, antiviral, anti-inflammatory, and hepatotoxic effects; and potential as a selective anticancer agent for colorectal cancer (Klein Gebbinck et al., 2002; Milutinović et al., 2019; Candela et al., 2020). *Teucrium chamaedrys*, or wall germander, is a woody shrub native to the Mediterranean region and is one of the most frequently cited *Teucrium* species in folk medicine (Jarić et al., 2020). It is specifically recognized in ethnobotanical studies for treating a wide variety of health issues, including digestive disorders, hypertension, and malaria (Pieroni et al., 2004; di Tizio et al., 2012; Arı et al., 2015; Jarić et al., 2020).

The medicinal properties of plants are typically a consequence of their specialized metabolite profiles. Ajugoideae—*Teucrium* in particular—is well known for its abundance of diterpenoids (Dictionary of Natural Products 30.2). Generally, diterpenoids are formed by the sequential activity of two diterpene synthases (diTPSs). A class II diTPS (TPS-c) first catalyzes the proton-mediated cyclization of a 20-carbon isoprenoid diphosphate, usually geranylgeranyl diphosphate (GGDP). Then, a class I (often a TPS-e) diTPS cleaves the diphosphate, further modifying the diterpene structure. *Teucrium* is especially rich in clerodane-type diterpenoids (Li et al., 2016; Schlecht et al., 2024). Clerodane synthases typically generate a class II product, either (−)-kolavenyl diphosphate ((−)-KDP), iso-KDP, or more rarely, *cis-trans*-clerodienyl diphosphate. To date, characterized iso-KDP synthases have only been identified in Lamiaceae species, including *Ajuga reptans*, *Scutellaria barbata,* and *Scutellaria baicalensis* (Johnson et al., 2019; Qiu et al., 2023). (−)-KDP synthases have been characterized in *Salvia divinorum*, *Salvia splendens*, *Vitex agnus-castus*, *Callicarpa americana*, *S. barbata*, *S. baicalensis*, and *Tripterygium wilfordii* (Andersen-Ranberg et al., 2016; Hansen et al., 2017; Pelot et al., 2017; Heskes et al., 2018; Hamilton et al., 2020). Iso-KDP differs from (−)-KDP by the site of final deprotonation, placing the double bond along the 4,18 bond rather than the 3,4 bond (Figure 1). The third and most uncommon structure has exclusively been found in the monocot species *Panicum virgatum*. It is a *cis-trans*-clerodienyl diphosphate, which, while sharing the same final quenching as (−)-KDP, is a different stereoisomer (Pelot et al., 2018). A variety of clerodane-derived products have been characterized specifically from *T. chamaedrys*, including various neo-clerodanes, chamaedryosides A–C, Teucrin, and others (Figure 1; Bedir et al., 2003; Fiorentino et al., 2009; Sadeghi et al., 2022; Dictionary of Natural Products 30.2).

The diversity of plant natural products is often driven by gene duplication via several mechanisms. Duplications can significantly increase novel gene formation by dispersing selective pressure, thereby allowing an explosion of metabolic diversity (Ren et al., 2018). Such duplications can occur through tandem or segmental duplication, both of which copy a region locally and can be the result of unequal DNA crossover events (Achaz et al., 2000). Repeats can also be introduced via retrotransposition, recognizable by the lack of introns and the presence of nearby inverted repeats (Hughes et al., 2003; Field et al., 2011). However, the most radical duplication method is whole-genome duplication (WGD). It is estimated that around 35% of all extant angiosperm species are polyploids with a history of WGDs (Wood et al., 2009; Landis et al., 2018; Godden et al., 2019). Nearly two-thirds (65%) of annotated plant genes are duplicated, with most derived from WGD events (Panchy et al., 2016).

To better understand how polyploidy affects diTPSs and chemical diversity in *Teucrium*, we sequenced and assembled the large (3 Gbp) tetraploid genome of *T. chamaedrys.* A recent WGD has

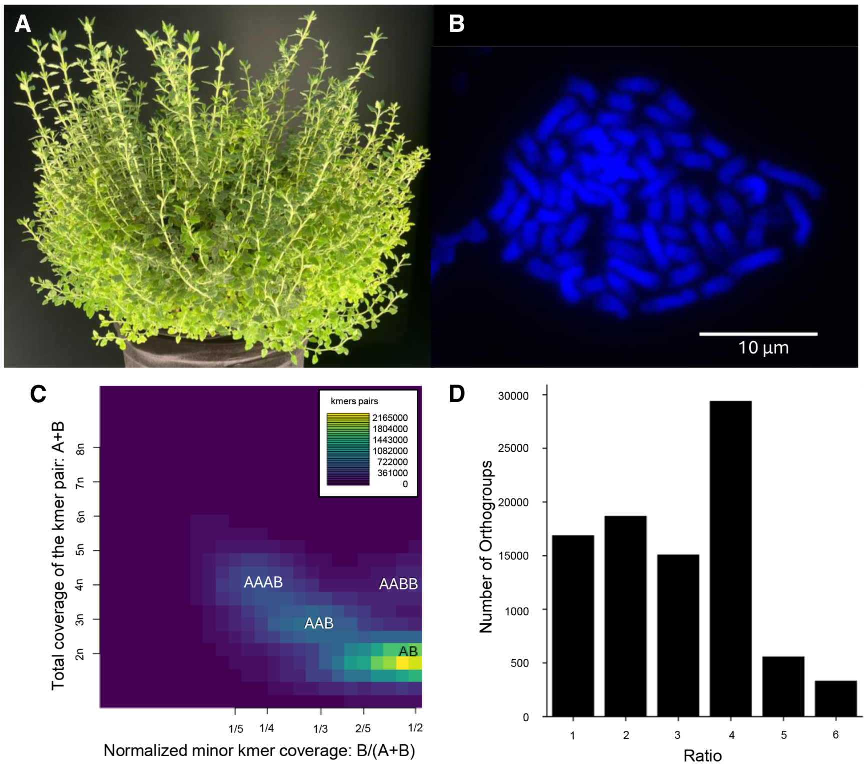

Figure 2. The tetraploid genome of *T. chamaedrys*.
(A) Image of mature *T. chamaedrys* shrub.
(B) A representative metaphase cell prepared from a root tip.
(C) Smudgeplot analysis showing evidence for genome duplication, with k-mers present at 4n configurations AAAB and AABB.
(D) Orthogroup proportions between *T. chamaedrys* and *A. thaliana*. Approximately 3000 orthogroups have four times as many orthologs in *T. chamaedrys* as in *A. thaliana.*

quadrupled TPSs in *T. chamaedrys*, including four copies of a large gene cluster containing the majority of the diTPSs. This cluster is also conserved in the closely related species *Teucrium marum* and predates the WGD, with diterpene chemistries in characterized species distinct from the clerodanes (Smit et al., 2024). The physical clustering of these diTPSs creates one of the largest biosynthetic gene clusters (BGCs) to date, spanning around 500 Kbp, within the range of the the 2 Mbp cluster present in *Ginkgo biloba* and the 580 Kbp cluster in opium poppy (Forman et al., 2022; Guo et al., 2018). Since *Teucrium* species are well known for their clerodane-derived products, we functionally characterized all four putative clerodane synthases in *T. chamaedrys*, along with a representative synthase from *Teucrium canadense*. Using comparative genomic, phylogenetic, and biochemical methods, we present the genetic underpinning and distinct evolution of two classes of diterpenoid chemistries within this species.

RESULTS AND DISCUSSION

T. chamaedrys genome reveals evidence of tetraploidy

To create a high-quality genome assembly for *T. chamaedrys*, we generated 265 Gbp of long reads using Oxford Nanopore Technology and 95 Gbp of short reads with Illumina sequencing. GenomeScope estimated the *T. chamaedrys* genome size at approximately 1.7 Gbp with low heterozygosity (0.14%; Supplemental Figure 1). Assembly, polishing, and removal of contigs shorter than 10 Kbp resulted in 3162 contigs (Supplemental Table 1) with a final assembly size of 2.9 Gbp (Supplemental Figure 2). Benchmarking Universal Single-Copy Orthologs (BUSCO; Manni et al., 2021) analysis with 2326 total BUSCO genes (eudicots_odb10) revealed 2274 (97.8%) complete orthologs, of which 60 (2.6%) were single copy, 2214 (95.2%) were duplicated, 8 (0.3%) were fragmented, and 44 (1.9%) were missing. Annotation of protein-coding genes identified 128 111 high-confidence genes. BUSCO analysis of the annotation revealed a similar set of statistics, with 2210 (95.0%) complete orthologs, 88 (3.8%) single copies, 2122 (91.2%) duplicated, 21 (0.9%) fragmented, and 95 (4.1%) missing. Overall, this demonstrates a high-quality assembly and annotation of the *T. chamaedrys* genome.

The presence of a highly duplicated BUSCO score suggests a recent WGD event, which is additionally corroborated by Smudgeplot k-mer analysis of genome duplication (Figure 2C; Ranallo-Benavidez et al., 2020). Polyploids frequently have highly divergent subgenomes, which can lead to an underestimation of shared k-mers (Supplemental Figure 3; Ranallo-Benavidez et al., 2020). The presence of a smudge at the "AAAB" position, coupled with the trace presence of "AABB," produced a strong signal at 4n coverage, indicating that *T. chamaedrys* is a tetraploid. This is consistent with OrthoFinder (Emms and Kelly, 2019) analysis comparing *T. chamaedrys* to *Arabidopsis thaliana* (*A. thaliana),* which revealed a predominant 4:1 ratio of orthologs, with weaker evidence for 2:1 and 3:1 ratios (Figure 2D). Furthermore, a chromosome count in dividing root tip cells and comparison to the closely related diploid relative *T. marum* (2n = 34; Smit et al., 2024) revealed that the majority of metaphase cells contained 2n = 62 for *T. chamaedrys* (Figure 2B), which is also consistent with a recent WGD event leading to tetraploidy. Previous chromosome counting efforts have shown this species to be variable in chromosome number (2n = 32–96; Ranjbar et al., 2018); the genome k-mer analysis, orthology, and cytogenetic evidence together support tetraploidy.

The evolutionary split between *T. marum* and *T. chamaedrys* is estimated at approximately 4 million years ago (Salmaki et al., 2016), suggesting that the WGD event within the *T. chamaedrys* lineage is relatively recent. Meiotic abnormalities, cell architecture changes, and genetic instability are among the detrimental side effects of WGD (Osborn et al., 2003; De Storme and Mason, 2014; Wang et al., 2021; Blasio et al., 2022), and many polyploids undergo re-diploidization to mitigate these effects (Li et al., 2021; Wang et al., 2021). This recent tetraploid genome may be a fleeting snapshot capturing

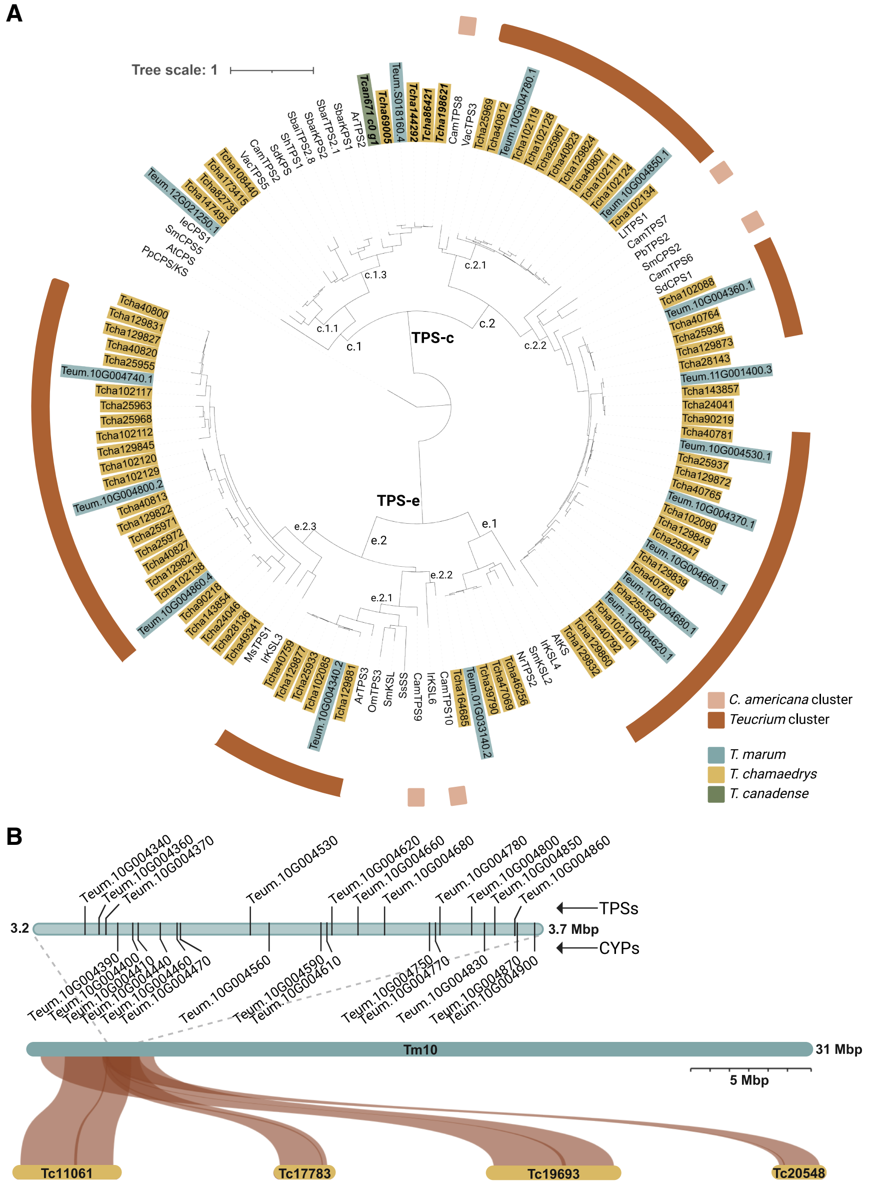

Figure 3. Phylogenetic analysis of the diterpene gene content in three *Teucrium* species.

(A) This tree is rooted by the class II/class I bifunctional *ent*-kaurene synthase from *Physcometrium patens*. Genes from *T. chamaedrys* are in gold, *T. marum* in blue, and *T. canadense* in green. Those without highlights are previously characterized diTPSs from other Lamiaceae species and *A. thaliana*. Bolded genes were functionally characterized in this study. Red and pink rings denote physical clustering in the genome of *Teucrium* and *C. americana*, respectively. Clades are labeled according to Johnson et al. (2019). Figure was created with iTOL and BioRender.com.

(B) Syntenic analysis between closely related *T. marum* (blue) and *T. chamaedrys* (gold) show a 1:4 syntenic relationship in a genomic region containing the majority of diTPSs genes. Inset shows the TPSs and CYPs (cytochromes P450) present in the *T. marum* cluster. Designated *T. chamaedrys* nomenclature (3A), Tca40(___), Tca12(___), Tca20(___), and Tca10(___), individually clustered TPS in four loci; (3B) Tc (_____), Tm10 corresponding to individual contigs. Figure was created with SynVisio and BioRender.com.

one of the many polyploidization events that are widespread across plant lineages. Therefore, the data we provide may inform future studies on the effects of polyploidization.

Phylogenetic evidence shows clustering and expansion of diTPSs in *Teucrium*

We estimated the phylogenetic relationships among 90 putative diTPS sequences in three *Teucrium* species (*T. chamaedrys*, *T. marum*, and *T. canadense*) alongside a set of functionally characterized diTPSs from other species in the Lamiaceae family and *A. thaliana* (Supplemental Table 2). One locus in *T. marum* (*Teum. 10G004340.2–Teum.10G004860.4*) accounts for 11 of the 15 predicted diTPSs. Similarly, four loci within *T. chamaedrys* (*Tcha40759–Tcha40827*, *Tcha129821–Tcha129881*, *Tcha25933–Tcha25972*, and *Tcha102085–Tcha102138*) account for 53 of the 74 predicted diTPSs, and they cluster across the phylogeny with corresponding orthologs in *T. marum* (Figure 3A).

There is clear synteny between the four genomic regions harboring these diTPSs in *T. chamaedrys* and the corresponding region in *T. marum* (Figure 3B), where *Tcha40759–Tcha40827* are located on contig Tc20548, *Tcha129821–Tcha129881* are on Tc17783, *Tcha25933–Tcha25972* are on Tc11061, and *Tcha102085–Tcha102138* are on Tc19693. This syntenic region contains predicted enzymes that include both class II and class I mechanisms, which is evidence for a large BGC. Interestingly, these clustered genes appear to be part of a Lamiaceae-wide miltiradiene-producing BGC (Bryson et al., 2023), as the clustered *Teucrium* genes are in the same phylogenetic clade (Supplemental Figures 4 and 5). Additionally, this BGC contains around 15 predicted CYPs from the CYP71 clan, which are often involved in diterpenoid metabolism and are also present in the Lamiaceae-wide BGC (Supplemental Figure 6). This *T. marum* cluster appears to form one of the largest diTPS BGCs to date, spanning around 500 Kbp.

Introducing genetic redundancy can lead to diversity in specialized metabolic pathways by relieving selective pressure (Ohno, 1970; Birchler and Yang, 2022). The high number of diTPS sequences in *T. chamaedrys* and *T. marum* reflects a major expansion of specialized metabolism (Figure 3A). *Teucrium* is among the top five Lamiaceae genera in unique diterpene skeleton production (Johnson et al., 2019), and the sheer number of predicted diTPSs in *T. chamaedrys* supports this. Phylogenetic blooms in a species can be attributed to tandem

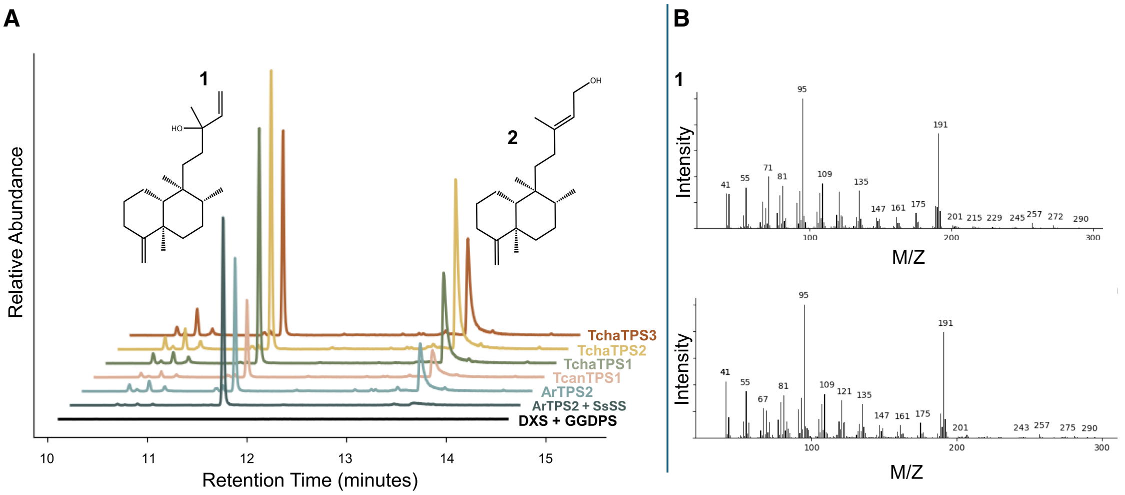

Figure 4. Extracted ion chromatogram (191 *m/z*) demonstrating iso-kolavenyl diphosphate synthase activity.

(A) Extracted ion chromatograms were stacked and shifted to facilitate comparison of products. Tested enzymes TchaTPS1, TchaTPS2, TchaTPS3, and TcanTPS1 were compared to the known iso-KDP synthase, ArTPS2, and the negative control, DXS+GGDPS. DXS+GGDPS is present in all samples. The peak at ~11.5 min corresponds to iso-kolavelool (1), and the peak at ~13.5 min corresponds to iso-kolavenol (2).

(B) Representative mass spectra of ArTPS2 corresponding to iso-kolavelool (1) and iso-kolavenol (2) peaks. Mass spectra of all relevant peaks are provided in Supplemental Figure 9. A representative chromatogram of three replicates is shown. Compound identification, level 1 (authentic standard, retention time, fragmentation pattern, *m/z*, high-resolution GC–MS data, Supplemental Table 4).

duplication and neofunctionalization, which appears to be the case here, with the large majority of diTPSs appearing to predate the speciation of *T. chamaedrys* and *T. marum* and the WGD event, further increasing the number present in *T. chamaedrys*. The abundance of diTPS sequences in *Teucrium* illustrates the vast diversity of diterpenoids harbored in these species, especially *T. chamaedrys*. The sequences from *T. canadense* were derived from transcriptomic rather than genomic data and therefore may not show a complete picture of diTPS diversity in this species. A higher proportion of diTPSs present in *T. chamaedrys* further suggests WGD, and syntenic analysis corroborates this.

Most plant species have two diTPSs that biosynthesize the initial pathway toward gibberellic acid: one in the phylogenetic clade TPS-e.1 and one in TPS-c.1, corresponding to class I and class II enzymes, respectively. The same 1:4 ratio observed in the BGC is seen in gibberellic acid synthesis genes as well (Figure 3A; TPS-c.1.1 and TPS-e.1). Where one TPS-e.1 is usually seen, there are four in *T. chamaedrys*, and the same is true for TPS-c.1 (Figure 3A). Given the haploid nature of the *T. marum* genome assembly (Smit et al., 2024), this 1:4 ratio is consistent with a WGD event present specifically in the lineage of *T. chamaedrys*.

Biochemical analysis reveals the basis of clerodane metabolism in *T. chamaedrys*

To better understand clerodane representation in *Teucrium*, we investigated the enzyme activity of four predicted clerodane synthase homologs in *T. chamaedrys* and one in *T. canadense*: TchaTPS1, TchaTPS2, TchaTPS3, and TcanTPS1. The fourth predicted clerodane synthase homolog from *T. chamaedrys* was determined to be inactive, with low expression in the plant (Tcha144292; Supplemental Figure 7). To functionally characterize each putative clerodane synthase, we used an *Agrobacterium*-mediated *Nicotiana benthamiana* transformation system in direct comparison with relevant published reference enzymes. We also co-expressed each enzyme with sclareol synthase (SsSS), a promiscuous class I diTPS that, in this context, produces exclusively iso-kolavelool from iso-KDP (Caniard et al., 2012). This allowed us to determine that the product at 11.5 min (1) was iso-kolavelool (neo-cleroda-4(18), 14-dien-13-ol), and the other major product at 13.5 min (2) was iso-kolavenol, based on comparison with the reference class II enzyme ArTPS2 (Figure 4A; Supplemental Figure 8; Johnson et al., 2019; mass spectra for 1 and 2 are given in Figure 4B). The mixture of products in runs without SsSS occurs as a result of dephosphorylation catalyzed by non-specific endogenous enzymes in *N. benthamiana* (Supplemental Figure 9). SsSS specifically produces iso-kolavalool as opposed to promiscuous cleavage by the endogenous *N. benthamiana* enzymes. Therefore, all active enzymes were found to produce iso-KDP, and none of the enzymes yielded conclusive evidence of (−)-KDP.

The presence of iso-KDP synthases in *T. chamaedrys* is not surprising, given that an evolutionarily close relative, *A. reptans*, possesses an ortholog, ArTPS2. Additionally, various *Teucrium* furanoclerodanes have been reported with a 4,18 double bond, 4,18 epoxides, and C18 esters that lack a C3–C4 double bond, features that presumably come from an iso-KDP precursor. All reported *T. chamaedrys* clerodanes are heavily modified and lack either the 3,4 or the 4,18 double bond but do have various C18 ester linkages (Dictionary of Natural Products 30.2), suggesting that they are likely formed by an iso-KDP precursor.

While we found no evidence of a dedicated (−)-KDP synthase in *T. chamaedrys*, some Lamiaceae species do contain (−)-KDP synthases and accumulate furanoclerodanes, including *Teucrium* species with a 3,4 double bond (Dictionary of Natural Products 30.2). Given the presence of iso-KDP-derived

chemistries in *T. chamaedrys*, either there has been a loss of the (−)-KDP enzyme in certain lineages or specific amino acid substitutions in the enzyme alter the products. Deprotonation of C3, rather than C18, would most likely require a slight positional shift, on the order of a few angstroms, of the base-acting residue to alter which proton is abstracted. It has been shown that blocking the deprotonation site of an *ent*-copalyl diphosphate synthase with a single amino acid substitution can convert the *ent-copalyl diphosphate* synthase into a (−)-KDP synthase (Potter et al., 2016). A third possibility is that another unrelated TPS-c may have convergently evolved (−)-KDP synthase activity.

This study represents the first functional characterization of diTPSs in *Teucrium*, laying the groundwork for future characterization of enzymes involved in subsequent steps of diterpenoid metabolism, such as clerodane-derived compounds like Teucrin, chamaedrosides, and neo-clerodanes. Understanding the natural biosynthetic pathways of these medicinally relevant compounds provides an important first step toward the biotechnological production and utilization of these terpenes in medicine and beyond.

METHODS

Plant growth conditions, tissue collection, and storage

The *T. chamaedrys* plant was purchased from Mountain Valley Growers (California, USA) and grown in a greenhouse. For DNA extraction, the plant was dark adapted for 72 h prior to harvesting. Healthy, mature leaves were collected, flash frozen in liquid nitrogen, and stored at −80°C. For RNA extraction, healthy, mature leaves and thoroughly rinsed roots were collected, flash frozen in liquid nitrogen, and stored at −80°C.

Nucleotide isolation

High-molecular-weight genomic DNA was extracted from *T. chamaedrys* leaves using a modified CTAB-based protocol (cetyltrimethylammonium bromide) (Li et al., 2020; Longley et al., 2023). Briefly, frozen tissue was ground into a fine powder with a mortar and pestle in liquid nitrogen and resuspended in a nuclear isolation buffer. After nuclei were isolated, CTAB was added, and high-molecular-weight nucleic acids were extracted with chloroform and isoamyl alcohol, washed with isopropanol, and treated with RNase (Thermo Fisher Scientific, MA, USA). Genomic DNA for short-read sequencing was extracted using the DNeasy Plant Mini Kit (QIAGEN, Hilden, Germany).

Library preparation and sequencing

DNA libraries for long-read sequencing with Nanopore (Oxford Nanopore Technologies, USA) were prepared using the Oxford Nanopore SQK-LSK114 Ligation Sequencing Kit v.14, and the library was loaded onto a PromethION FLO-PRO114M (R10.4.1) flow cell. Prior to long-read sequencing, the DNA was processed with the Standard Short Read Eliminator Kit (Circulomics, MD, USA). MinKNOW (v.22.10.07) was used for sequencing control, and base calling was performed with Guppy (v.6.3.9) using the high-accuracy model.

For short-read sequencing, library preparation was performed using the Roche Kapa HyperPrep DNA Library Kit with Unique Dual Index adapters (Sigma-Aldrich, MO, USA). The completed library was assessed for quality and quantified using Qubit dsDNA HS, Agilent 4200 TapeStation HS DNA1000, and Invitrogen Collibri Illumina Library Quantification qPCR assays. The sample was loaded onto one lane of an Illumina v.1.5 S4 flow cell using the Xp Workflow. Sequencing was performed in a 2× 150 bp paired-end format using a NovaSeq 6000 (v.1.5) 300 cycle reagent cartridge (Illumina, CA, USA). Base calling was performed using Illumina Real Time Analysis (v.3.4.4), and output from the Real Time Analysis software was demultiplexed and converted to FASTQ format with Illumina's Bcl2fastq (v.2.20.0).

Genome size and heterozygosity estimation

Jellyfish (v.2.3.0; Marçais and Kingsford, 2011) was used to estimate genome size and heterozygosity via k-mer analysis. Trimmed and filtered 31-mers from the Illumina DNA libraries were used.

Ploidy analysis

KMC (Kokot et al., 2017) was also used to count k-mers in the genome using a k-mer length of 31, yielding 4 876 867 453 unique k-mers. K-mer analysis was visualized using GenomeScope (v.1.0; Supplemental Figure 1; Vurture et al., 2017). Subsequently, ploidy was measured using Smudgeplot analysis (Ranallo-Benavidez et al., 2020). The lower coverage threshold cutoff was set to 12, and the upper cutoff was set to 2,800, estimated using "cutoff" from the Smudgeplot suite. The Smudgeplot analysis output was hand-annotated according to Ranallo-Benavidez et al. (2020), as the original output did not include the AAAB annotation (Figure 2; Supplemental Figure 3).

Genome assembly

Raw Nanopore DNA reads with mean Q-scores greater than 7 were used and processed with Porechop (v.0.2.4) to remove adapters, Chopper (v.0.8.0-0; De Coster et al., 2018) to filter reads shorter than 10 Kb, and Filtlong (v.0.2.0) to remove the worst 10% of reads based on quality. Sequences were then assembled using Flye (v.2.9; Kolmogorov et al., 2019) with a minimum overlap of 5 Kbp, two iterations of polishing, and haplotype retention enabled. The draft assembly was polished once using Medaka (v.1.4.3) and the model "r1041_e82_400bps_hac_g632." BWA-MEM2 (v.2.0; Vasimuddin and et al., 2019) was used to align the Illumina paired-end reads to the draft assembly for error correction. The resulting draft assembly was polished with one round of Pilon (v.1.24; Walker et al., 2014) using the "diploid" option. Contigs smaller than 100 Kbp were then removed from the assembly. To eliminate potential contamination, Kraken2 (v.2.1.3; Wood et al., 2019) was used with the database "PlusPFP" (https://benlangmead.github.io/aws-indexes/k2). Approximately 0.44% of the assembly was determined to be human and subsequently removed. No further contamination was detected.

Genome annotation

The draft genome was first mined for *de novo* repeats using Repeat Modeler (v.2.0.2a; Flynn et al., 2020). These *de novo* repeats, along with Viridiplantae repeats from RepBase, were used by Repeat Masker (v.4.1.1; Chen, 2004) to mask the draft genome. Next, RNA sequencing data from *T. chamaedrys* (SRA: PRJNA1124528) mature leaves and roots were aligned to the draft genome using HISAT2 (v.2.1.0; Kim et al., 2019). In addition to this transcript evidence, protein evidence from the closely related species *T. marum* (Smit et al., 2024) was used as an input for BRAKER (v.2.1.6; Altschul et al., 1990; Stanke et al., 2006; 2008; Camacho et al., 2009; Quinlan, 2014; Kovaka et al., 2019; Pertea and Pertea, 2020; Gabriel et al., 2021; Bruna et al., 2023) with the flag "–etpmode" to create initial gene models. These gene models were then fed into MAKER (Law et al., 2015), along with RNA sequencing evidence and protein evidence from *A. thaliana* (TAIR v.11; Cheng et al., 2017) and *T. marum* (Smit et al., 2024), to create a working gene model set (Supplemental Table 3).

This yielded 217 373 working gene models, which were later filtered down to 128 111 high-confidence gene models. Of the original 217 373 gene models, 153 810 had an annotation edit distance score of less than one and/or contained a protein domain, indicating evidence for transcripts or protein homology (Yandell and Ence, 2012). Of those, we kept one gene model per locus, yielding 144 380 gene models. Although a repeat-masked genome was used initially, we found additional transposable element-related genes, which, when removed, yielded 134 486 gene models. All genes shorter than 300 bp were then removed,

leaving 128 264 gene models. Finally, we removed non-plant contamination according to Kraken2 (Wood et al., 2019) for a final high-confidence gene model count of 128 111.

Chromosome counting

Root tips were harvested from greenhouse-grown rooted cuttings and pretreated with nitrous oxide at a pressure of 160 psi (approximately 10.9 atm) for 40 min. Subsequently, the root tips were fixed in a solution of three parts ethanol to one part acetic acid and maintained at 22°C until enzymatic treatment. An enzymatic solution containing 4% cellulase (Yakult Pharmaceutical, Tokyo, Japan), 2% pectinase (Plant Media, Dublin, OH, USA), and 2% pectolyase (Sigma Chemical, St. Louis, MO, USA) was used to digest the root tips for 50 min at 37°C. Chromosomes were prepared using a stirring method as described by Xin et al. (2020) and counterstained with 4′,6-diamidino-2-phenylindole in VectaShield antifade solution (Vector Laboratories, Burlingame, CA, USA). Images were captured with a QImaging Retiga EXi Fast 1394 CCD camera (Teledyne Photometrics, Tucson, AZ, USA) attached to an Olympus BX51 epifluorescence microscope. Image processing was performed using Meta Imaging Series 7.5 software, and the final image contrast was adjusted using Adobe Photoshop (Adobe, San Jose, CA, USA). Chromosome counting was conducted on at least 10 metaphase spreads.

Phylogeny

T. canadense reads were downloaded from the NCBI Sequence Read Archive (SRA) database (SRR5150734), and the split read files were assembled into a *de novo* transcriptome using Trinity (v.2.9.1; Grabherr et al., 2011). The resulting mRNA was filtered for the longest open reading frame and translated into protein sequences using TransDecoder (v.2.1.0; Haas et al., 2013; https://github.com/TransDecoder/TransDecoder). *T. marum* gene models were downloaded from Figshare (https://figshare.com/articles/dataset/Teucrium_marum_genome_assembly/25109411). The representative gene models from each of the three *Teucrium* species were queried using BLAST (BLAST+ v.2.13.0, e value = 1e−20; Camacho et al., 2009) against a bait set of 34 functionally characterized diTPSs (Supplemental Table 2). Resulting protein matches were identified and combined with the bait set. Multiple sequence alignments were generated using ClustalOmega (v.1.2.4; Sievers et al., 2011), and phylogenetic trees were generated using RAxML using the model "protgammaauto," algorithm "a," and 100 bootstrap replicates (v.8.2.12; Stamatakis, 2014).

Synteny

The BLAST function makeblastdb (e value = 1e−10, 5 alignments) was used to create protein databases for *T. chamaedrys* and *T. marum* (Smit et al., 2024). Syntenic analysis was performed using the standard MCScanX pipeline (match score = 50; match size = 5; gap penalty = −1; overlap window = 5; e value = 1e−5; max gaps = 25; Wang et al., 2012). Results were visualized using SynVisio (Bandi and Gutwin, 2020).

Cloning and transient expression

Candidate enzymes from *T. chamaedrys* were synthesized (Twist Bioscience, CA, USA) and cloned into the plant expression vector pEAQ-HT (Sainsbury et al., 2009) for use in transient expression in *N. benthamiana*. Sequences were validated via Sanger sequencing. *N. benthamiana* plants were grown for 4–5 weeks in a controlled growth room under a 12-h light and 12-h dark (22°C) cycle before infiltration. Coexpression constructs were transformed separately into *Agrobacterium tumefaciens* strain LBA4404. Cultures were grown overnight at 30°C in lysogeny broth containing 50 μg/ml kanamycin and 50 μg/ml rifampicin. Cultures were collected by centrifugation and washed twice with approximately 10 ml water before being resuspended and diluted to an OD600 of 1.0 in water with 200 μM acetosyringone. Cultures were incubated at 30°C for 1–2 h, after which equal volumes of each culture were mixed for each combination of enzymes. *N. benthamiana* leaves were infiltrated on the underside (abaxial side) with a 1-ml syringe. All gene constructs were co-infiltrated with two genes encoding rate-limiting steps in the upstream 2-C-methyl-D-erythritol 4-phosphate pathway, *Plectranthus barbatus* 1-deoxy-D-xylulose-5-phosphate synthase and GGDP synthase, to boost production of the diterpene precursor GGDP (Andersen-Ranberg et al., 2016). Plants were returned to the controlled growth room for 5 days. Approximately 200 mg of fresh weight from three separate infiltrated leaves was extracted with 1.5 ml hexane overnight at room temperature. Plant material was collected by centrifugation, and the organic phase was removed for gas chromatography–mass spectrometry (GC–MS) analysis.

GC–MS analysis

All GC–MS analyses were performed on an Agilent 7890 A GC with an Agilent VF-5ms column (30 m × 250 μm × 0.25 μm, with 10 m EZ-Guard) and an Agilent 5975 C detector. The inlet was set to 250°C with splitless injection of 1 μl using He carrier gas (flow rate = 1 ml/min). The detector was activated following a 4-min solvent delay. All assays and tissue analyses used the following method: temperature ramp start 40°C, hold 1 min, 40°C/min to 200°C, hold 4.5 min, 20°C/min to 240°C, 10°C/min to 280°C, 40°C/min to 320°C, and hold 5 min. The MS scan range was set to 40–400.

DATA AND CODE AVAILABILITY

The data supporting the findings of this work are available within the paper and supplemental information. Raw genomic WGS and FLcDNA (full-length cDNA) reads generated in this study have been deposited in the NCBI SRA under accession number SRA: PRJNA1246154. Sequences for the four functionally characterized enzymes are available in the NCBI BankIt under accession numbers PQ246887–PQ246890. The Genome assembly, annotation, raw GC–MS, and a list of *Teucrium* sequences used in Figure 3 are available through our Dryad Repository (https://doi.org/10.5061/dryad.4mw6m90kp). A voucher specimen of *T. chamaedrys* has been deposited at the Michigan State University Herbarium and can be found under catalog number MSC0291921 and secondary catalog number 415574.

FUNDING

A.E.B. and N.S. would like to acknowledge the generous support of the Neogen Land Grant Prize, an endowed grant program administered by the Office of Research and Innovation at Michigan State University (MSU), which supports graduate students in translating their research into real-world applications that positively impact society and the US economy. A.E.B. and D.M. are funded by a National Science Foundation (NSF)-IMPACTS Training Grant (DGE-1828149). A.E.B., D.M., and B.H. are funded by NSF Dimensions of Biodiversity (DEB 1737898). N.S. is supported by the National Institute of General Medical Sciences of the National Institutes of Health under award number T32 GM110523. B.H. and N.S. gratefully acknowledge the US Department of Energy Great Lakes Bioenergy Research Center Cooperative Agreement DE-SC0018409. B.H. also acknowledges startup funding from the Department of Biochemistry and Molecular Biology at MSU and support from AgBioResearch (MICL02454), as well as a generous endowment from James K. Billman, Jr., MD. B.H. is also supported in part by the National Science Foundation under Grant number 1737898. C.R.B. acknowledges funding from the University of Georgia, the Georgia Research Alliance, and Georgia Seed Development. J.J. acknowledges support from the NSF under grant number ISO-2029959.

ACKNOWLEDGMENTS

We would like to thank Matt Chansler, Jennifer S. Apland, and Alan Prather for processing the herbarium specimen; Emily R. Lanier for RNA extraction; Britta Hamberger for plant care; and Patrick Edger and Jim Leebens-Mack for discussions. This work was supported in part by computational resources and services provided by the Institute for Cyber-Enabled Research at MSU, and by resources and technical expertise from the Georgia Advanced Computing Resource Center, a partnership between the Office of the Vice President for Research and the Office of the Vice President for Information Technology at the University of

Georgia. We thank the MSU RTSF Genomics Core for sequencing services and the MSU Mass Spectrometry and Metabolomics Core Facility for access to the GC–MS instrumentation. MSU occupies the ancestral, traditional, and contemporary lands of the Anishinaabeg–Three Fires Confederacy of Ojibwe, Odawa, and Potawatomi peoples. MSU resides on land ceded in the 1819 Treaty of Saginaw. Any opinions, findings, and conclusions or recommendations expressed in this material are those of the authors and do not necessarily reflect the views of the National Science Foundation. The content is solely the responsibility of the authors and does not necessarily represent the official views of the National Institutes of Health. No conflict of interest declared.

AUTHOR CONTRIBUTIONS

Conceptualization, A.E.B., N.S., and B.H.; investigation and methodology, A.E.B., D.M., K.L.C., and J.P.H.; genomic analysis, A.E.B. and D.M.; phylogenetic analysis and biochemical assays, A.E.B.; chromosome squash, H.X.; writing – original draft, all authors; writing – review & editing, A.E.B. and B.H.; funding acquisition, A.E.B., N.S., and B.H.; resources, B.H., J. J., and C.R.B.; supervision, J.J., C.R.B., and B.H.

SUPPLEMENTAL INFORMATION

Supplemental information is available at *Plant Communications Online*.

Received: September 4, 2024
Revised: January 14, 2025
Accepted: May 30, 2025
Published: June 3, 2025

REFERENCES

Achaz, G., Coissac, E., Viari, A., and Netter, P. (2000). Analysis of Intrachromosomal Duplications in Yeast *Saccharomyces cerevisiae*: A Possible Model for Their Origin. Mol. Biol. Evol. **17**:1268–1275. https://doi.org/10.1093/oxfordjournals.molbev.a026410.

Altschul, S.F., Gish, W., Miller, W., Myers, E.W., and Lipman, D.J. (1990). Basic local alignment search tool. J. Mol. Biol. **215**:403–410. https://doi.org/10.1016/S0022-2836(05)80360-2.

Andersen-Ranberg, J., Kongstad, K.T., Nielsen, M.T., Jensen, N.B., Pateraki, I., Bach, S.S., Hamberger, B., Zerbe, P., Staerk, D., Bohlmann, J., et al. (2016). Expanding the landscape of diterpene structural diversity through stereochemically controlled combinatorial biosynthesis. Angew. Chem. **55**:2142–2146. https://doi.org/10.1002/anie.201510650.

Arı, S., Temel, M., Kargıoğlu, M., and Konuk, M. (2015). Ethnobotanical survey of plants used in Afyonkarahisar-Turkey. J. Ethnobiol. Ethnomed. **11**:1–15. https://doi.org/10.1186/s13002-015-0067-6.

Bandi, V., and Gutwin, C. (2020). SynVisio: An interactive multiscale synteny visualization tool for MCScanX. In In Proceedings of the 46th Graphics Interface Conference on Proceedings of Graphics Interface 2020 (GI'20). Interactive Exploration of Genomic Conservation, Waterloo, CAN: Canadian Human-Computer Communications Society. https://synvisio.github.io/#/.

Bedir, E., Manyam, R., and Khan, I.A. (2003). *Neo*-clerodane diterpenoids and phenylethanoid glycosides from *Teucrium chamaedrys* L. Phytochemistry **63**:977–983. https://doi.org/10.1016/S0031-9422(03)00378-9.

Birchler, J.A., and Yang, H. (2022). The multiple fates of gene duplications: Deletion, hypofunctionalization, subfunctionalization, neofunctionalization, dosage balance constraints, and neutral variation. Plant Cell **34**:2466–2474. https://doi.org/10.1093/plcell/koac076.

Blasio, F., Prieto, P., Pradillo, M., and Naranjo, T. (2022). Genomic and Meiotic Changes Accompanying Polyploidization. Plants **11**:125. https://doi.org/10.3390/plants11010125.

Bruna, T., Lomsadze, A., and Borodovsky, M. (2023). GeneMark-ETP: Automatic Gene Finding in Eukaryotic Genomes in Consistence with Extrinsic Data. Preprint at bioRxiv. https://doi.org/10.1101/2023.01.13.524024.

Bryson, A.E., Lanier, E.R., Lau, K.H., Hamilton, J.P., Vaillancourt, B., Mathieu, D., Yocca, A.E., Miller, G.P., Edger, P.P., Buell, C.R., et al. (2023). Uncovering a miltiradiene biosynthetic gene cluster in the Lamiaceae reveals a dynamic evolutionary trajectory. Nat. Commun. **14**:343. https://doi.org/10.1038/s41467-023-35845-1.

Camacho, C., Coulouris, G., Avagyan, V., Ma, N., Papadopoulos, J., Bealer, K., and Madden, T.L. (2009). BLAST+: Architecture and applications. BMC Bioinf. **10**:421. https://doi.org/10.1186/1471-2105-10-421.

Candela, R.G., Rosselli, S., Bruno, M., and Fontana, G. (2021). A Review of the Phytochemistry, Traditional Uses and Biological Activities of the Essential Oils of Genus *Teucrium*. Planta Med. **87**:432–479. https://doi.org/10.1055/a-1293-5768.

Caniard, A., Zerbe, P., Legrand, S., Cohade, A., Valot, N., Magnard, J. L., Bohlmann, J., and Legendre, L. (2012). Discovery and functional characterization of two diterpene synthases for sclareol biosynthesis in *Salvia sclarea* (L.) and their relevance for perfume manufacture. BMC Plant Biol. **12**:119. https://doi.org/10.1186/1471-2229-12-119.

Chen, N. (2004). Using RepeatMasker to Identify Repetitive Elements in Genomic Sequences. Curr. Protoc. Bioinformatics **Chapter 4**, Unit 410–4.10.14. https://doi.org/10.1002/0471250953.bi0410s05.

Cheng, C.-Y., Krishnakumar, V., Chan, A.P., Thibaud-Nissen, F., Schobel, S., and Town, C.D. (2017). Araport11: a complete reannotation of the *Arabidopsis thaliana* reference genome. Plant J. **89**:789–804. https://doi.org/10.1111/tpj.13415.

De Coster, W., D'Hert, S., Schultz, D.T., Cruts, M., and Van Broeckhoven, C. (2018). NanoPack: visualizing and processing long-read sequencing data. Bioinformatics (Oxford, England) **34**:2666–2669. https://doi.org/10.1093/bioinformatics/bty149.

De Storme, N., and Mason, A. (2014). Plant speciation through chromosome instability and ploidy change: Cellular mechanisms, molecular factors and evolutionary relevance. Curr. Plant Bio. **1**:10–33. https://doi.org/10.1016/j.cpb.2014.09.002.

Dictionary of Natural Products 30.2 (no date). Available at: https://dnp.chemnetbase.com/faces/chemical/ChemicalSearch.xhtml (Accessed: 11 March 2022).

Emms, D.M., and Kelly, S. (2019). OrthoFinder: phylogenetic orthology inference for comparative genomics. Genome Biol. **20**:238. https://doi.org/10.1186/s13059-019-1832-y.

Field, B., Fiston-Lavier, A.S., Kemen, A., Geisler, K., Quesneville, H., and Osbourn, A.E. (2011). Formation of plant metabolic gene clusters within dynamic chromosomal regions. Proc. Natl. Acad. Sci. USA **108**:16116–16121. https://doi.org/10.1073/pnas.1109273108.

Fiorentino, A., D'Abrosca, B., Esposito, A., Izzo, A., Pascarella, M.T., D'Angelo, G., and Monaco, P. (2009). Potential allelopathic effect of neo-clerodane diterpenes from *Teucrium chamaedrys* (L.) on stenomediterranean and weed cosmopolitan species. Biochem. Systemat. Ecol. **37**:349–353. https://doi.org/10.1016/j.bse.2009.06.006.

Flynn, J.M., Hubley, R., Goubert, C., Rosen, J., Clark, A.G., Feschotte, C., and Smit, A.F. (2020). RepeatModeler2 for automated genomic discovery of transposable element families. Proc. Natl. Acad. Sci. USA **117**:9451–9457. https://doi.org/10.1073/pnas.1921046117.

Forman, V., Luo, D., Geu-Flores, F., Lemcke, R., Nelson, D.R., Kampranis, S.C., Staerk, D., Møller, B.L., and Pateraki, I. (2022). A gene cluster in Ginkgo biloba encodes unique multifunctional cytochrome P450s that initiate ginkgolide biosynthesis. Nat. Commun. **13**:5143. https://doi.org/10.1038/s41467-022-32879-9.

Gabriel, L., Hoff, K.J., Brůna, T., Borodovsky, M., and Stanke, M. (2021). TSEBRA: transcript selector for BRAKER. BMC Bioinf. **22**:566. https://doi.org/10.1186/s12859-021-04482-0.

Godden, G.T., Kinser, T.J., Soltis, P.S., and Soltis, D.E. (2019). Phylotranscriptomic analyses reveal asymmetrical gene duplication dynamics and signatures of ancient polyploidy in mints. Genome Biol. Evol. **11**:3393–3408. https://doi.org/10.1093/gbe/evz239.

Grabherr, M.G., Haas, B.J., Yassour, M., Levin, J.Z., Thompson, D.A., Amit, I., Adiconis, X., Fan, L., Raychowdhury, R., Zeng, Q., et al. (2011). Trinity: reconstructing a full-length transcriptome without a genome from RNA-Seq data. Nat. Biotechnol. **29**:644–652. https://doi.org/10.1038/nbt.1883.

Guo, L., Winzer, T., Yang, X., Li, Y., Ning, Z., He, Z., Teodor, R., Lu, Y., Bowser, T.A., Graham, I.A., and Ye, K. (2018). The opium poppy genome and morphinan production. Science **362**:343–347. https://doi.org/10.1126/science.aat4096.

Haas, B.J., Papanicolaou, A., Yassour, M., Grabherr, M., Blood, P.D., Bowden, J., Couger, M.B., Eccles, D., Li, B., Lieber, M., et al. (2013). De novo transcript sequence reconstruction from RNA-seq using the Trinity platform for reference generation and analysis. Nat Protoc **8**:1494–1512. https://doi.org/10.1038/nprot.2013.084.

Hamilton, J.P., Godden, G.T., Lanier, E., Bhat, W.W., Kinser, T.J., Vaillancourt, B., Wang, H., Wood, J.C., Jiang, J., Soltis, P.S., et al. (2020). Generation of a chromosome-scale genome assembly of the insect-repellent terpenoid-producing Lamiaceae species, *Callicarpa americana*. GigaScience **9**:giaa093. https://doi.org/10.1093/gigascience/giaa093.

Hansen, N.L., Heskes, A.M., Hamberger, B., Olsen, C.E., Hallström, B. M., Andersen-Ranberg, J., and Hamberger, B. (2017). The terpene synthase gene family in *Tripterygium wilfordii* harbors a labdane-type diterpene synthase among the monoterpene synthase TPS-b subfamily. Plant J. **89**:429–441. https://doi.org/10.1111/tpj.13410.

Heskes, A.M., Sundram, T.C.M., Boughton, B.A., Jensen, N.B., Hansen, N.L., Crocoll, C., Cozzi, F., Rasmussen, S., Hamberger, B., Hamberger, B., et al. (2018). Biosynthesis of bioactive diterpenoids in the medicinal plant *Vitex agnus-castus*. Plant J. **93**:943–958. https://doi.org/10.1111/tpj.13822.

Hughes, A.L., Friedman, R., Ekollu, V., and Rose, J.R. (2003). Non-random association of transposable elements with duplicated genomic blocks in *Arabidopsis thaliana*. Mol. Phylogenet. Evol. **29**:410–416. https://doi.org/10.1016/S1055-7903(03)00262-8.

Jarić, S., Mitrović, M., and Pavlović, P. (2020). Ethnobotanical Features of *Teucrium* Species. In Teucrium Species: Biology and Applications, M. Stanković, ed. (Springer International Publishing), pp. 111–142. https://doi.org/10.1007/978-3-030-52159-2_5.

Johnson, S.R., Bhat, W.W., Bibik, J., Turmo, A., Hamberger, B., Evolutionary Mint Genomics Consortium, and Hamberger, B. (2019). A database-driven approach identifies additional diterpene synthase activities in the mint family (Lamiaceae). J. Biol. Chem. **294**:1349–1362. https://doi.org/10.1074/jbc.RA118.006025.

Kim, D., Paggi, J.M., Park, C., Bennett, C., and Salzberg, S.L. (2019). Graph-based genome alignment and genotyping with HISAT2 and HISAT-genotype. Nat. Biotechnol. **37**:907–915. https://doi.org/10.1038/s41587-019-0201-4.

Klein Gebbinck, E.A., Jansen, B.J.M., and de Groot, A. (2002). Insect antifeedant activity of clerodane diterpenes and related model compounds. Phytochemistry **61**:737–770. https://doi.org/10.1016/S0031-9422(02)00174-7.

Kokot, M., Długosz, M., and Deorowicz, S. (2017). KMC 3: counting and manipulating k-mer statistics. Bioinformatics **33**:2759–2761. https://doi.org/10.1093/bioinformatics/btx304.

Kolmogorov, M., Yuan, J., Lin, Y., and Pevzner, P.A. (2019). Assembly of long, error-prone reads using repeat graphs. Nat. Biotechnol. **37**:540–546. https://doi.org/10.1038/s41587-019-0072-8.

Kovaka, S., Zimin, A.V., Pertea, G.M., Razaghi, R., Salzberg, S.L., and Pertea, M. (2019). Transcriptome assembly from long-read RNA-seq alignments with StringTie2. Genome Biol. **20**:278. https://doi.org/10.1186/s13059-019-1910-1.

Landis, J.B., Soltis, D.E., Li, Z., Marx, H.E., Barker, M.S., Tank, D.C., and Soltis, P.S. (2018). Impact of whole-genome duplication events on diversification rates in angiosperms. Am. J. Bot. **105**:348–363. https://doi.org/10.1002/ajb2.1060.

Law, M., Childs, K.L., Campbell, M.S., Stein, J.C., Olson, A.J., Holt, C., Panchy, N., Lei, J., Jiao, D., Andorf, C.M., et al. (2015). Automated Update, Revision, and Quality Control of the Maize Genome Annotations Using MAKER-P Improves the B73 RefGen_v3 Gene Models and Identifies New Genes. Plant Physiol. **167**:25–39. https://doi.org/10.1104/pp.114.245027.

Li, R., Morris-Natschke, S.L., and Lee, K.-H. (2016). Clerodane diterpenes: sources, structures, and biological activities. Nat. Prod. Rep. **33**:1166–1226. https://doi.org/10.1039/c5np00137d.

Li, Z., McKibben, M.T.W., Finch, G.S., Blischak, P.D., Sutherland, B.L., and Barker, M.S. (2021). Patterns and Processes of Diploidization in Land Plants. Annu. Rev. Plant Biol. **72**:387–410. https://doi.org/10.1146/annurev-arplant-050718-100344.

Li, Z., Parris, S., and Saski, C.A. (2020). A simple plant high-molecular-weight DNA extraction method suitable for single-molecule technologies. Plant Methods **16**:38. https://doi.org/10.1186/s13007-020-00579-4.

Longley, R., Robinson, A., Liber, J.A., Bryson, A.E., Morales, D.P., LaButti, K., Riley, R., Mondo, S.J., Kuo, A., Yoshinaga, Y., et al. (2023). Comparative genomics of Mollicutes-related endobacteria supports a late invasion into Mucoromycota fungi. Commun. Biol. **6**:948. https://doi.org/10.1038/s42003-023-05299-8.

Manni, M., Berkeley, M.R., Seppey, M., Simão, F.A., and Zdobnov, E. M. (2021). BUSCO update: Novel and streamlined workflows along with broader and deeper phylogenetic coverage for scoring of eukaryotic, prokaryotic, and viral genomes. Mol. Biol. Evol. **38**:4647–4654. https://doi.org/10.1093/molbev/msab199.

Marçais, G., and Kingsford, C. (2011). A fast, lock-free approach for efficient parallel counting of occurrences of k-mers. Bioinformatics **27**:764–770. https://doi.org/10.1093/bioinformatics/btr011.

Menichini, F., Conforti, F., Rigano, D., Formisano, C., Piozzi, F., and Senatore, F. (2009). Phytochemical composition, anti-inflammatory and antitumour activities of four *Teucrium* essential oils from Greece. Food Chem. **115**:679–686. https://doi.org/10.1016/j.foodchem.2008.12.067.

Milutinović, M.G., Maksimović, V.M., Cvetković, D.M., Nikodijević, D. D., Stanković, M.S., Pešić, M., and Marković, S.D. (2019). Potential of *Teucrium chamaedrys* L. to modulate apoptosis and biotransformation in colorectal carcinoma cells. J. Ethnopharmacol. **240**:111951. https://doi.org/10.1016/j.jep.2019.111951.

Ohno, S. (1970). Duplication for the Sake of Producing More of the Same. In Evolution by Gene Duplication, S. Ohno, ed. (Springer), pp. 59–65. https://doi.org/10.1007/978-3-642-86659-3_11.

Osborn, T.C., Pires, J.C., Birchler, J.A., Auger, D.L., Chen, Z.J., Lee, H. S., Comai, L., Madlung, A., Doerge, R.W., Colot, V., et al. (2003). Understanding mechanisms of novel gene expression in polyploids. Trends Genet. **19**:141–147. https://doi.org/10.1016/S0168-9525(03)00015-5.

Panchy, N., Lehti-Shiu, M., and Shiu, S.-H. (2016). Evolution of Gene Duplication in Plants. Plant Physiol. **171**:2294–2316. https://doi.org/10.1104/pp.16.00523.

Pelot, K.A., Mitchell, R., Kwon, M., Hagelthorn, L.M., Wardman, J.F., Chiang, A., Bohlmann, J., Ro, D.K., and Zerbe, P. (2017). Biosynthesis of the psychotropic plant diterpene salvinorin A: Discovery and characterization of the *Salvia divinorum* clerodienyl diphosphate synthase. Plant J. **89**:885–897. https://doi.org/10.1111/tpj.13427.

Pelot, K.A., Chen, R., Hagelthorn, D.M., Young, C.A., Addison, J.B., Muchlinski, A., Tholl, D., and Zerbe, P. (2018). Functional Diversity of Diterpene Synthases in the Biofuel Crop Switchgrass. Plant Physiol. **178**:54–71. https://doi.org/10.1104/pp.18.00590.

Pertea, G., and Pertea, M. (2020). GFF Utilities: GffRead and GffCompare. F1000Res. **9**:ISCB.Comm.J-304. https://doi.org/10.12688/f1000research.23297.2.

Pieroni, A., Quave, C.L., and Santoro, R.F. (2004). Folk pharmaceutical knowledge in the territory of the Dolomiti Lucane, inland southern Italy. J. Ethnopharmacol. **95**:373–384. https://doi.org/10.1016/j.jep.2004.08.012.

Potter, K.C., Zi, J., Hong, Y.J., Schulte, S., Malchow, B., Tantillo, D.J., and Peters, R.J. (2016). Blocking Deprotonation with Retention of Aromaticity in a Plant ent-Copalyl Diphosphate Synthase Leads to Product Rearrangement. Angew. Chem. Int. Ed. Engl. **55**:634–638. https://doi.org/10.1002/anie.201509060.

Qiu, T., Li, Y., Wu, H., Yang, H., Peng, Z., Du, Z., Wu, Q., Wang, H., Shen, Y., and Huang, L. (2023). Tandem duplication and subfunctionalization of clerodane diterpene synthase originate the blooming of clerodane diterpenoids in *Scutellaria barbata*. Plant J. **116**:375–388. https://doi.org/10.1111/tpj.16377.

Quinlan, A.R. (2014). BEDTools: The Swiss-Army Tool for Genome Feature Analysis. Curr. Protoc. Bioinformatics **47**:11.12.1–11.12.34. https://doi.org/10.1002/0471250953.bi1112s47.

Ranallo-Benavidez, T.R., Jaron, K.S., and Schatz, M.C. (2020). GenomeScope 2.0 and Smudgeplot for reference-free profiling of polyploid genomes. Nat. Commun. **11**:1432. https://doi.org/10.1038/s41467-020-14998-3.

Ranjbar, M., Mahmoudi, C., and Nazari, H. (2018). An overview of chromosomal criteria and biogeography in the genus *Teucrium* (Lamiaceae). Caryologia **71**:63–79. https://doi.org/10.1080/00087114.2017.1420587.

Ren, R., Wang, H., Guo, C., Zhang, N., Zeng, L., Chen, Y., Ma, H., and Qi, J. (2018). Widespread Whole Genome Duplications Contribute to Genome Complexity and Species Diversity in Angiosperms. Mol. Plant **11**:414–428. https://doi.org/10.1016/j.molp.2018.01.002.

Ritz, M., Ahmad, N., Brueck, T., and Mehlmer, N. (2023). Comparative Genome-Wide Analysis of Two *Caryopteris x Clandonensis* Cultivars: Insights on the Biosynthesis of Volatile Terpenoids. Plants **12**:632. https://doi.org/10.3390/plants12030632.

Sadeghi, Z., Yang, J.L., Venditti, A., and Moridi Farimani, M. (2022). A review of the phytochemistry, ethnopharmacology and biological activities of *Teucrium* genus (Germander). Nat. Prod. Res. **36**:5647–5664. https://doi.org/10.1080/14786419.2021.2022669.

Sainsbury, F., Thuenemann, E.C., and Lomonossoff, G.P. (2009). pEAQ: Versatile expression vectors for easy and quick transient expression of heterologous proteins in plants. Plant Biotechnol. J. **7**:682–693. https://doi.org/10.1111/j.1467-7652.2009.00434.x.

Salmaki, Y., Kattari, S., Heubl, G., and Bräuchler, C. (2016). Phylogeny of non-monophyletic *Teucrium* (Lamiaceae: Ajugoideae): Implications for character evolution and taxonomy. Taxon **65**:805–822. https://doi.org/10.12705/654.8.

Schlecht, N.J., Lanier, E.R., Andersen, T.B., Brose, J., Holmes, D., and Hamberger, B.R. (2024). CYP76BK1 orthologs catalyze furan and lactone ring formation in clerodane diterpenoids across the mint family. Plant J. **120**:984–997. https://doi.org/10.1111/tpj.17031.

Sievers, F., Wilm, A., Dineen, D., Gibson, T.J., Karplus, K., Li, W., Lopez, R., McWilliam, H., Remmert, M., Söding, J., et al. (2011). Fast, scalable generation of high-quality protein multiple sequence alignments using Clustal Omega. Mol. Syst. Biol. **7**:539. https://doi.org/10.1038/msb.2011.75.

Smit, S.J., Ayten, S., Radzikowska, B.A., Hamilton, J.P., Langer, S., Unsworth, W.P., Larson, T.R., Buell, C.R., and Lichman, B.R. (2024). The genomic and enzymatic basis for iridoid biosynthesis in cat thyme (*Teucrium marum*). Plant J. **118**:1589–1602. https://doi.org/10.1111/tpj.16698.

Stamatakis, A. (2014). RAxML version 8: A tool for phylogenetic analysis and post-analysis of large phylogenies. Bioinformatics **30**:1312–1313. https://doi.org/10.1093/bioinformatics/btu033.

Stanke, M., Keller, O., Gunduz, I., Hayes, A., Waack, S., and Morgenstern, B. (2006). AUGUSTUS: Ab initio prediction of alternative transcripts. Nucleic Acids Res. **34**:W435–W439. https://doi.org/10.1093/nar/gkl200.

Stanke, M., Diekhans, M., Baertsch, R., and Haussler, D. (2008). Using native and syntenically mapped cDNA alignments to improve de novo gene finding. Bioinformatics **24**:637–644. https://doi.org/10.1093/bioinformatics/btn013.

di Tizio, A., Łuczaj, Ł.J., Quave, C.L., Redžić, S., and Pieroni, A. (2012). Traditional food and herbal uses of wild plants in the ancient South-Slavic diaspora of Mundimitar/Montemitro (Southern Italy). J. Ethnobiol. Ethnomed. **8**:21. https://doi.org/10.1186/1746-4269-8-21.

Vasimuddin, M., Misra, S., Li, H., and Aluru, S. (2019). Efficient architecture-aware acceleration of BWA-MEM for multicore systems. In 2019 IEEE International Parallel and Distributed Processing Symposium (IPDPS), pp. 314–324. https://doi.org/10.1109/IPDPS.2019.00041.

Vurture, G.W., Sedlazeck, F.J., Nattestad, M., Underwood, C.J., Fang, H., Gurtowski, J., and Schatz, M.C. (2017). GenomeScope: fast reference-free genome profiling from short reads. Bioinformatics **33**:2202–2204. https://doi.org/10.1093/bioinformatics/btx153.

Walker, B.J., Abeel, T., Shea, T., Priest, M., Abouelliel, A., Sakthikumar, S., Cuomo, C.A., Zeng, Q., Wortman, J., Young, S. K., et al. (2014). Pilon: An Integrated Tool for Comprehensive Microbial Variant Detection and Genome Assembly Improvement. PLoS One **9**:e112963. https://doi.org/10.1371/journal.pone.0112963.

Wang, X., Morton, J.A., Pellicer, J., Leitch, I.J., and Leitch, A.R. (2021). Genome downsizing after polyploidy: mechanisms, rates and selection pressures. Plant J. **107**:1003–1015. https://doi.org/10.1111/tpj.15363.

Wang, Y., Tang, H., Debarry, J.D., Tan, X., Li, J., Wang, X., Lee, T.h., Jin, H., Marler, B., Guo, H., et al. (2012). MCScanX: A toolkit for detection and evolutionary analysis of gene synteny and collinearity. Nucleic Acids Res. **40**:e49. https://doi.org/10.1093/nar/gkr1293.

Wood, D.E., Lu, J., and Langmead, B. (2019). Improved metagenomic analysis with Kraken 2. Genome Biol. **20**:257. https://doi.org/10.1186/s13059-019-1891-0.

Wood, T.E., Takebayashi, N., Barker, M.S., Mayrose, I., Greenspoon, P.B., and Rieseberg, L.H. (2009). The frequency of polyploid speciation in vascular plants. Proc. Natl. Acad. Sci. USA **106**:13875–13879. https://doi.org/10.1073/pnas.0811575106.

Xin, H., Zhang, T., Wu, Y., Zhang, W., Zhang, P., Xi, M., and Jiang, J. (2020). An extraordinarily stable karyotype of the woody *Populus* species revealed by chromosome painting. Plant J. **101**:253–264. https://doi.org/10.1111/tpj.14536.

Yandell, M., and Ence, D. (2012). A beginner's guide to eukaryotic genome annotation. Nat. Rev. Genet. **13**:329–342. https://doi.org/10.1038/nrg3174.

Plant Communications, Volume *6*

Supplemental information

A high-quality genome assembly of the tetraploid *Teucrium chamaedrys* unveils a recent whole-genome duplication and a large biosynthetic gene cluster for diterpenoid metabolism

Abigail E. Bryson, Kevin L. Childs, Nicholas Schlecht, Davis Mathieu, John P. Hamilton, Haoyang Xin, Jiming Jiang, C. Robin Buell, and BjÖrn Hamberger

Supplemental Files

A high-quality genome assembly of the tetraploid *Teucrium chamaedrys* unveils a recent whole genome duplication and a large biosynthetic gene cluster for diterpenoid metabolism

Bryson *et al* 2025.

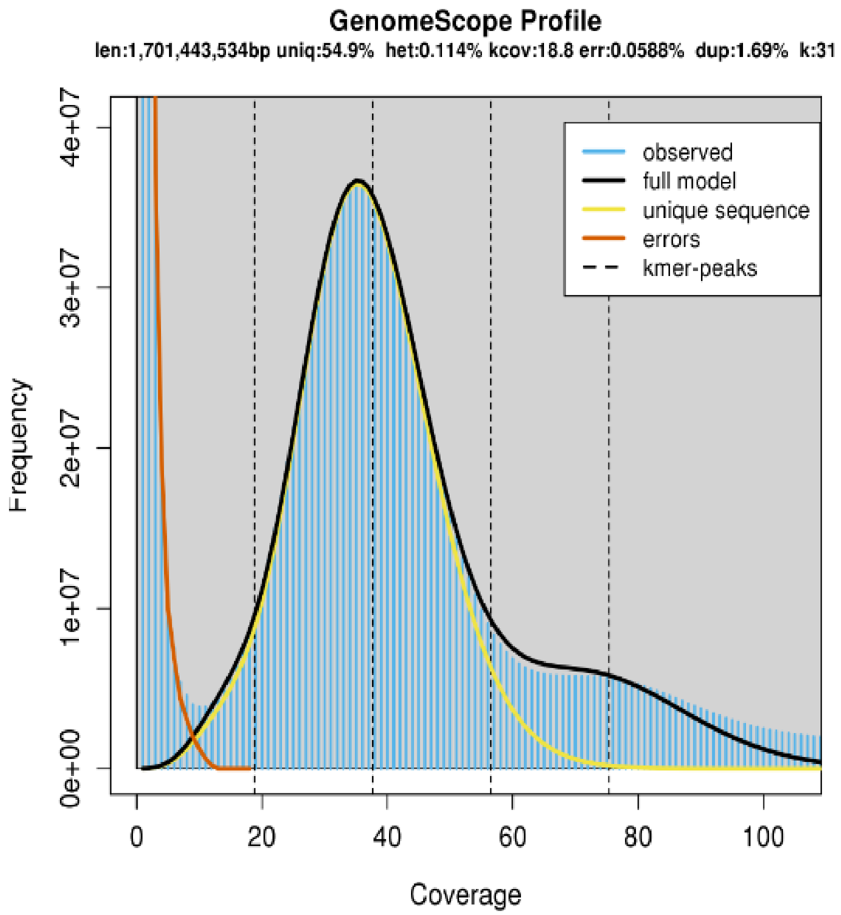


```
GenomeScope version 1.0
k = 31

property                      min               max
Heterozygosity                0.0889729%        0.138491%
Genome Haploid Length         1,680,787,676 bp  1,701,443,534 bp
Genome Repeat Length          758,478,916 bp    767,800,161 bp
Genome Unique Length          922,308,759 bp    933,643,373 bp
Model Fit                     90.0569%          97.8819%
Read Error Rate               0.0587909%        0.0587909%
```

Supplemental Figure 1. GenomeScope results. GenomeScope measures heterozygosity and predicts genome size based on short reads.

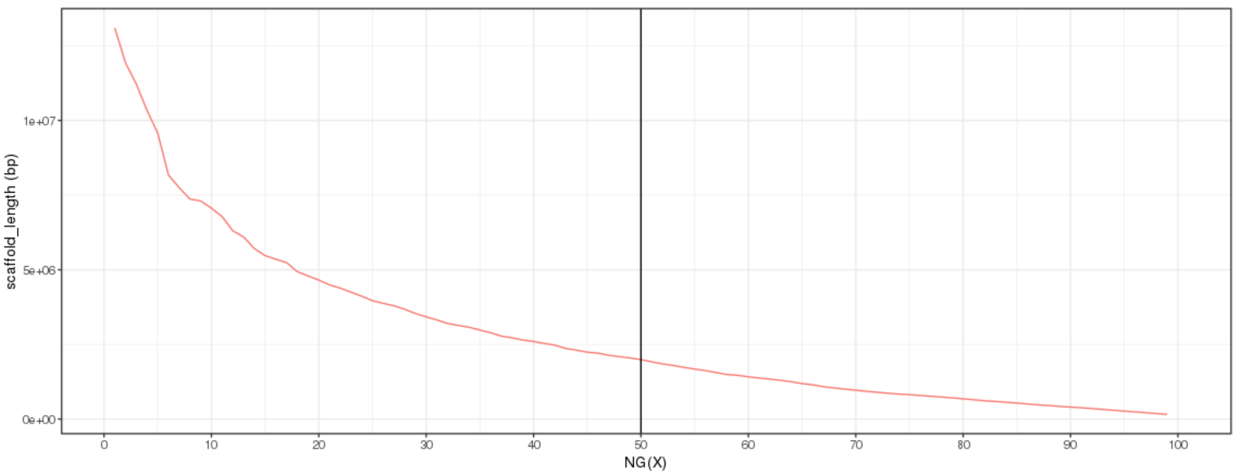

Supplemental Figure 2. Comparison of the genome assembly quality based on NG(X) values. Line represents contig lengths at different NG levels.

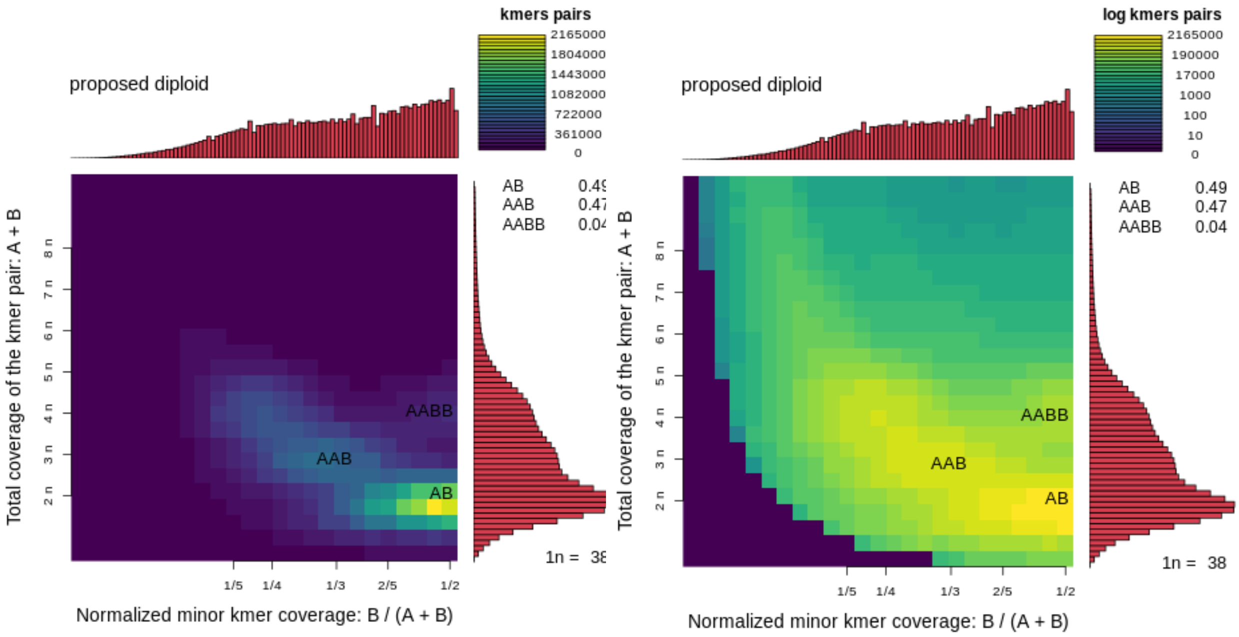

Supplemental Figure 3. Complete and unedited Smudgeplot results. Although this result proposed diploidy, evidence within this figure and across other analyses disagree. Since polyploids can have diverse genomes, sometimes k-mer based analysis can predict diploid for poplyploid species. An unmarked smudge at the 'AAAB' position coupled with the marked smudge at 'AABB' show evidence for 4n coverage of k-mers.

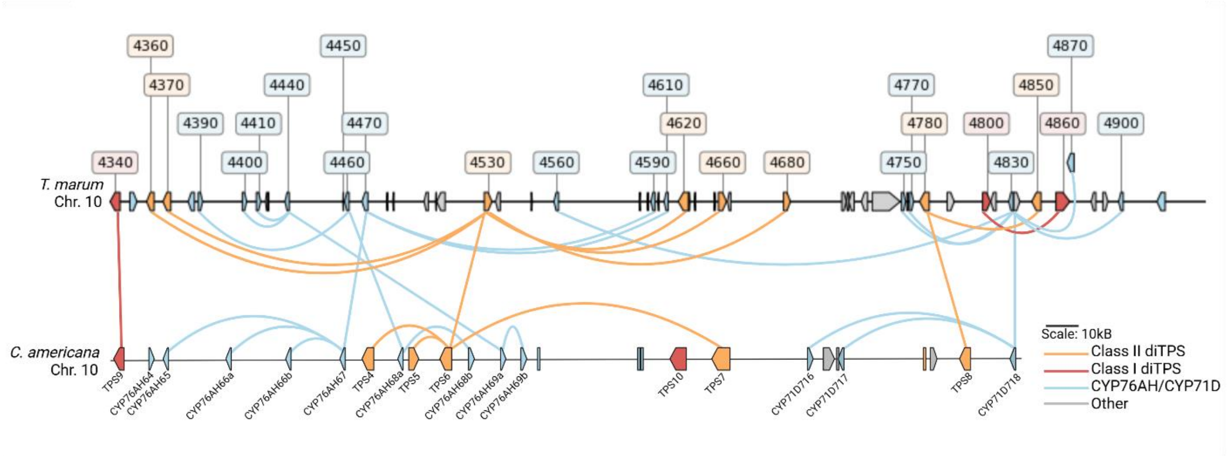

Supplemental Figure 4. Synteny between the BGC regions investigated in *T. marum* compared to *Callicarpa americana*. The top line shows the physical map of the region on chromosome 10 of *T. marum* investigated as a BGC in this study. The bottom line shows the syntenically equivalent region on chromosome 10 in *C. americana*. Synteny lines represent phylogenetic clades and can be referenced in Supplemental Figure 5.

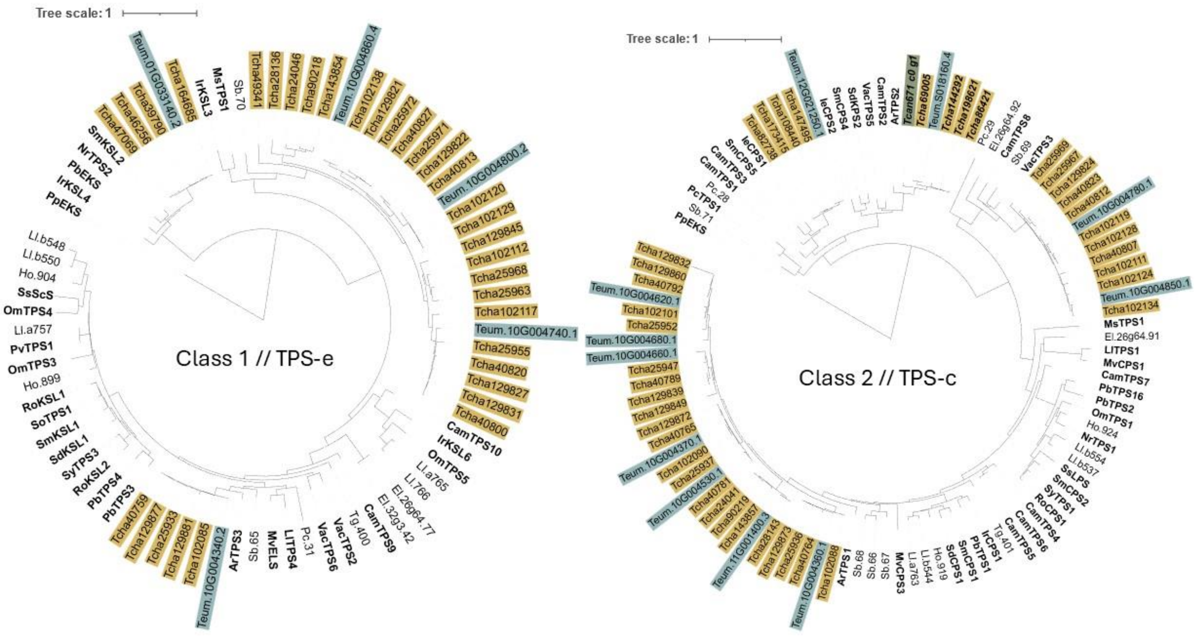

Supplemental Figure 5. Phylogenetics shows relationships of *Teucrium* diTPSs to other mint diTPSs present in a BGC. All *T. marum* (blue) and *T. chamaedrys* (gold) sequences with reference enzymes bolded in addition to sequences found to be part of the Lamiaceae-wide miltiradiene cluster reported in Bryson *et al* 2023. 100 BS confidence.

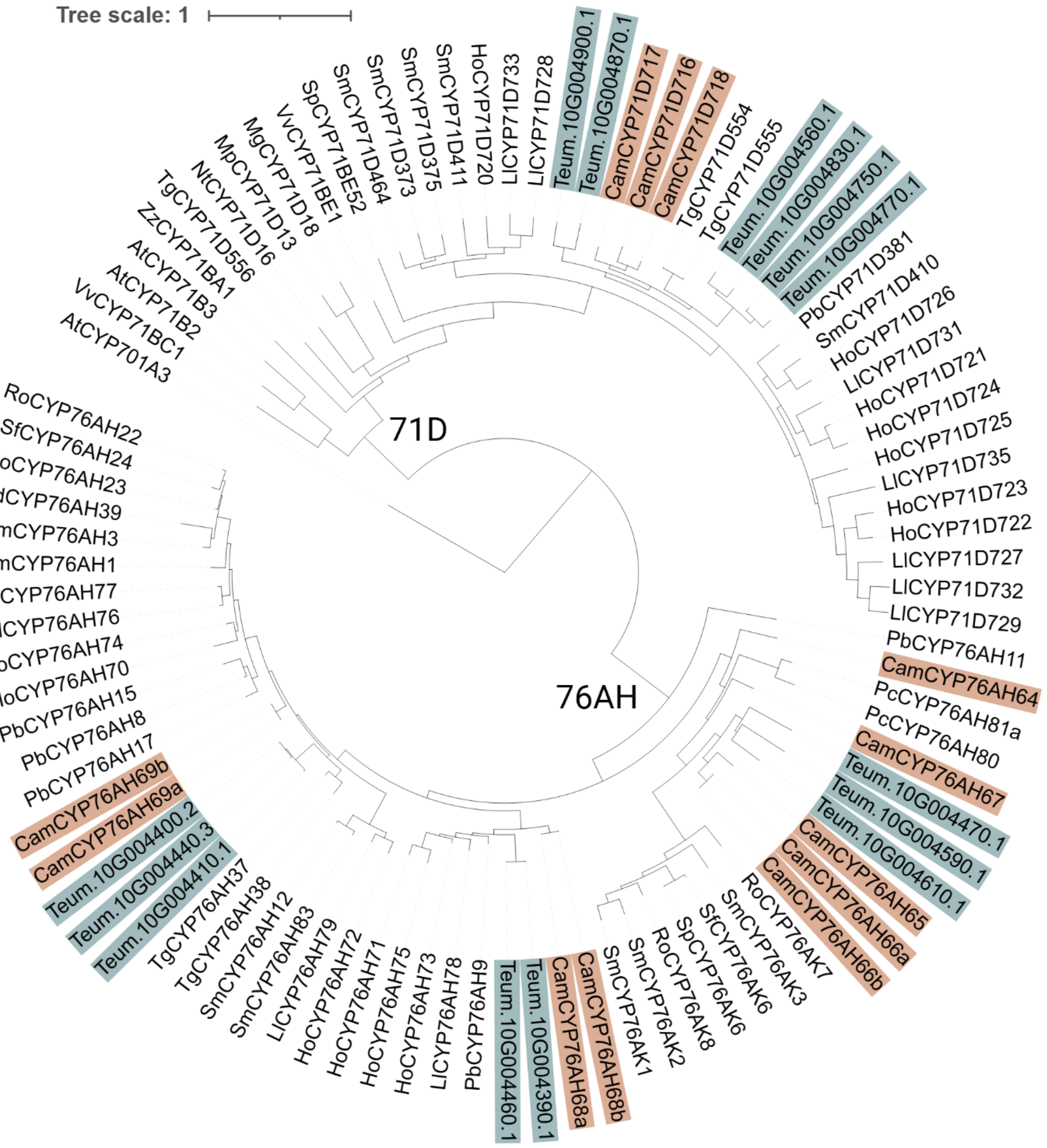

Supplemental Figure 6. Phylogenetics shows relationships of *Teucrium* CYPs to other mint CYPs present in a BGC. *T. marum* (blue) and *C. americana* (rose) sequences in addition to sequences found to be part of the Lamiaceae-wide miltiradiene cluster reported in Bryson *et al* 2023. 100 BS confidence.

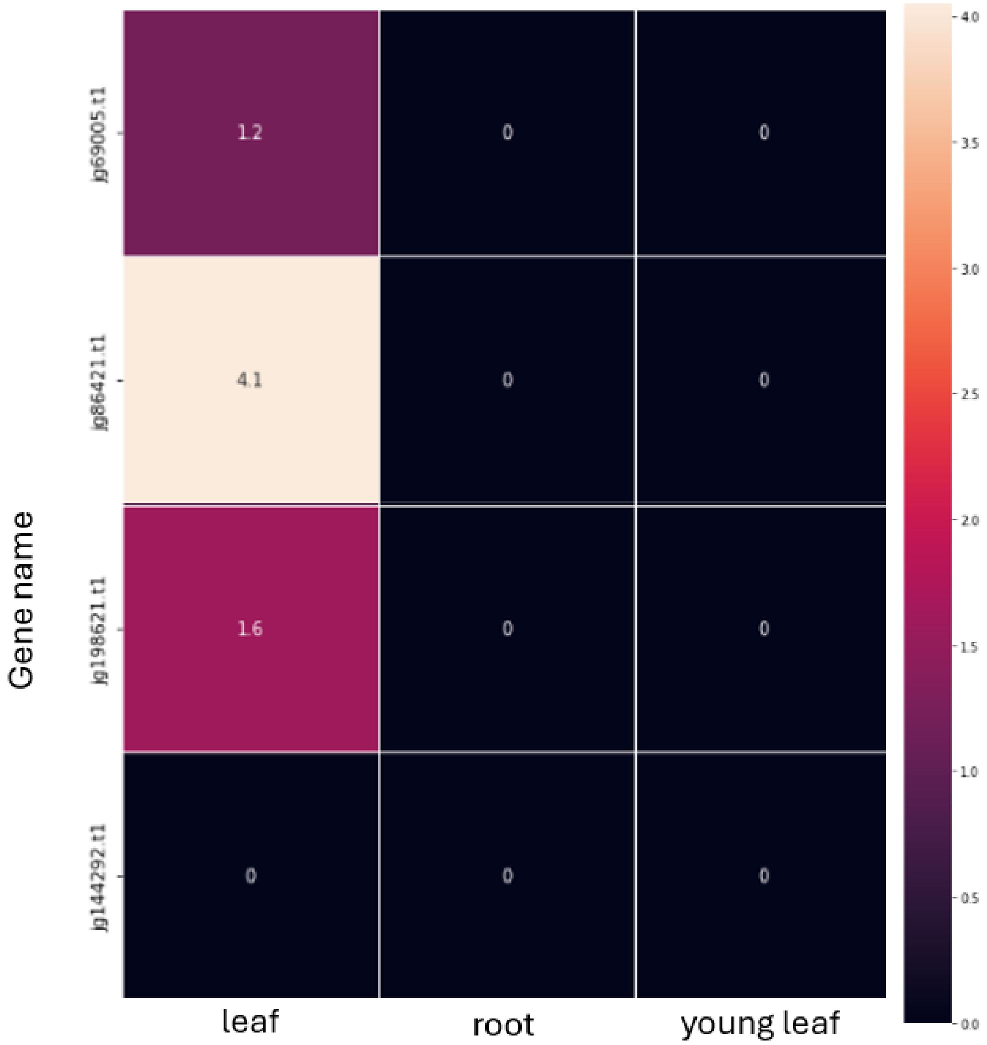

Supplemental Figure 7. RNA expression in *T. chamaedrys* tissues for putative clerodane synthases in this study. Genes characterized in this study only have expression in mature leaves.

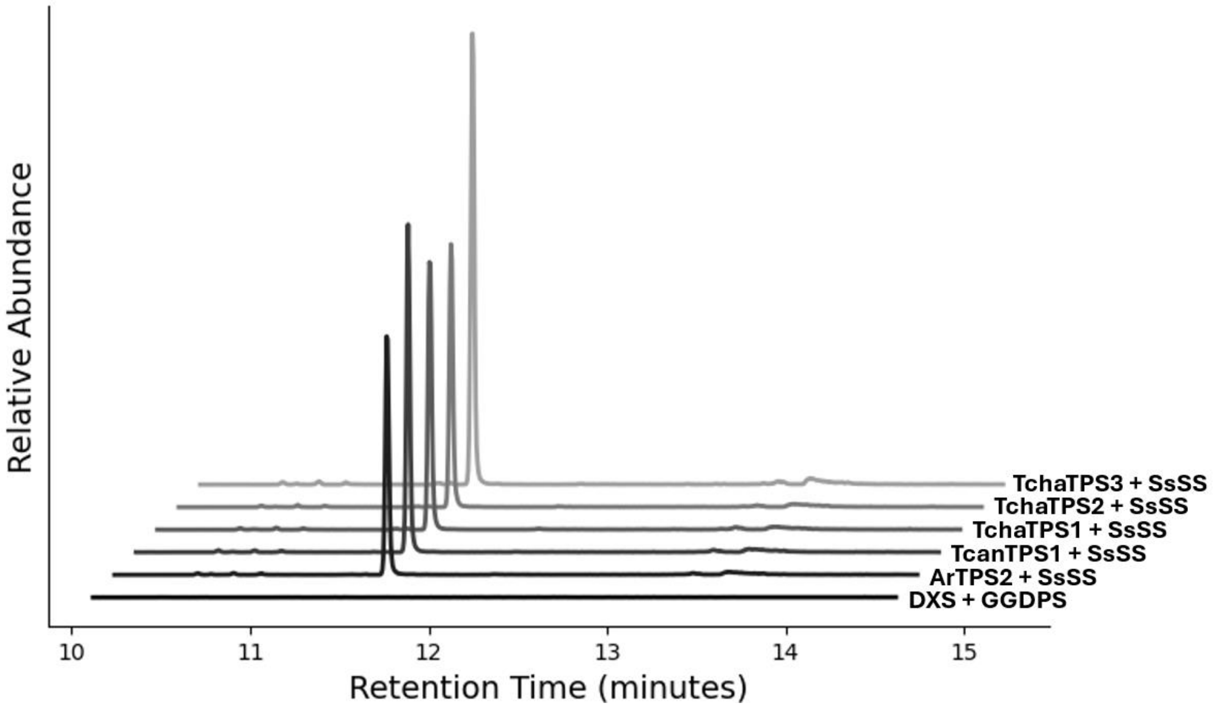

Supplemental Figure 8. Extracted ion chromatogram (191 m/z) demonstrating iso-kolavalool activity. Each EIC was stacked and shifted to compare their products. Peak indicates production of the KDP-derivative, iso-kolavalool. All samples have DXS+GGDPS even if not explicitly stated. Representative chromatograms of at least 3 replicates shown.

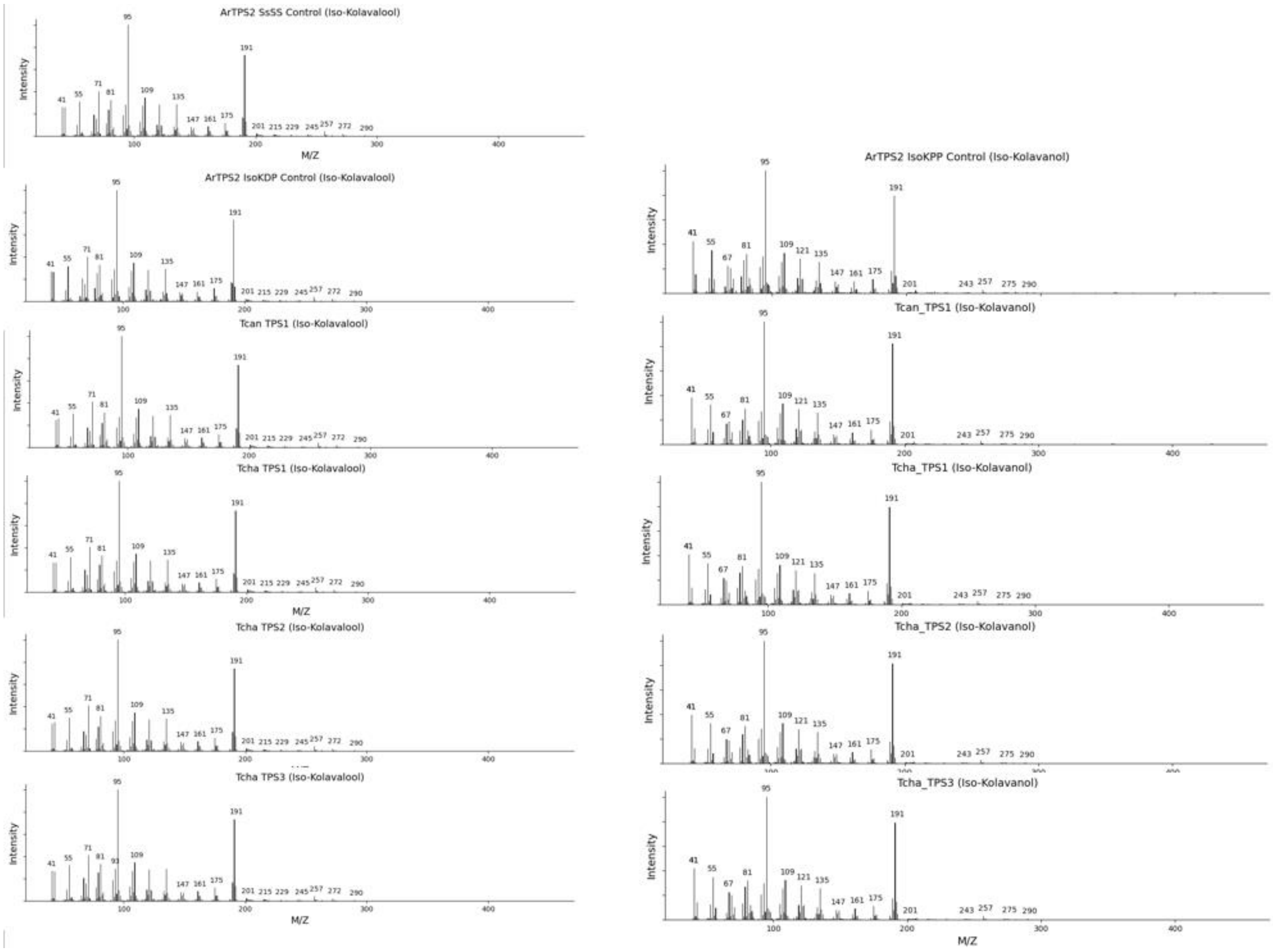

Supplemental Figure 9. Mass spectra for each peak shown in Figure 4. Left column corresponds to iso-kolavalool and the right column corresponds to iso-kolavanol. Both are KDP-derived products.

Total size (bp)	2,926,520,217
Number of contigs	3,148
Largest contig (bp)	16,924,500
N50	1,961,342
N90	385,309
L50	390
L90	1,777
GC Content	39.36%

Supplemental Table 1. Assembly statistics for the genome assembly of *Teucrium chamaedrys*.

Enzyme Name	Species	NCBI GenBank Number
PpCPS/KS	*Physcometrium patens*	BAF61135.1
AtCPS	*Arabidopsis thaliana*	Q38802.1
SmCPS5	*Salvia miltiorrhiza*	AEZ55692.1
IeCPS1	*Isodon eriocalyx*	G3E4M6.1
CamTPS2	*Callicarpa americana*	QMW69082.1
VacTPS5	*Vitex agnus-castus*	A0A2K9RG07.1
SdKPS	*Salvia divinorum*	A0A1S5RW73.1
ShTPS1	*Salvia hispanica*	XP_047942076.1
SbaiTPS2.8	*Scutellaria baicalensis*	UNZ93479.1
SbarKSP2	*Scutellaria barbata*	WJZ49120.1
SbarTPS2.1	*Scutellaria barbata*	UNZ11786.1
SbarKPS1	*Scutellaria barbata*	WJZ49119.1
ArTPS2	*Ajuga reptans*	AZB50378.1
VacTPS3	*Vitex agnus-castus*	A0A2K9RFZ8.1
LlTPS1	*Leonotis Leonurus*	AZB50381.1
CamTPS7	*Callicarpa americana*	UXG91343.1
CamTPS6	*Callicarpa americana*	QMW69083.1
PbTPS2	*Plectranthus barbatus*	AHW04047.1
SmCPS2	*Salvia miltiorrhiza*	AEZ55684.1
SdCPS1	*Salvia divinorum*	APH81399.1
AtKS	*Arabidopsis thaliana*	Q9SAK2.1
IrKSL4	*Isodon rubescens*	A0A1Z3GBK8.1
SmKSL2	*Salvia miltiorrhiza*	H6VLG5.2
NrTPS2	*Nepta racemosa*	AZB50370.1
CamTPS10	*Callicarpa americana*	UXG91345.1
IrKSL6	*Isodon rubescens*	A0A1Z3GCD1.1
CamTPS9	*Callicarpa americana*	UXG91344.1
SsSS	*Salvia sclerea*	G8GJ94.1
OmTPS3	*Origanum majorana*	AZB50371.1
SmSKL	*Salvia miltiorrhiza*	C8XPS0.1
ArTPS3	*Ajuga reptans*	AZB50367.1
IrKSL3	*Isodon rubescens*	A0A1X9ISH5.2
MsTPS1	*Mentha spicata*	AZB50369.1

Supplemental Table 2. Enzyme names, corresponding species, and accession number for all reference sequences used in generating the phylogeny in Figure 3.

Number of gene models	128,111
Average gene length (bp)	2,937.5
Average exons per gene model	4.4
Average exon length (bp)	223.8

Supplemental Table 3. Assembly statistics for the genome annotation of *Teucrium chamaedrys*.

Supplemental Table 4. High resolution mass spectrometry, GC-HRT+ GC/Time-of-Flight MS, given mass defect between predicted ions and measured mass. Details for methods, spectra and data are given in 'Data Availability'.

Sample	masslynx selected region for Calculated mass	masslynx subtracted region for Calculated mass	Predicted Structure	Ion Description	Predicted Monoisotopic mass	Measured mass	Δppm
DXS-GGPPS + ArTPS2	5524-5579	5354-5508 & 5625-5786	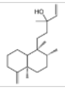	Parent ion	290.261	Below annotaiton threshold	NA
DXS-GGPPS + ArTPS2	5524-5579	5354-5508 & 5625-5786	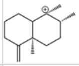	Second largest fragment (decalin core)	191.1794	191.1793	0.52
DXS-GGPPS + ArTPS2	5524-5579	5354-5508 & 5625-5786	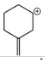	Largest fragment	95.0856	95.0854	2.10
DXS-GGPPS + ArTPS2 + SsSS	5524-5579	5354-5508 & 5625-5786	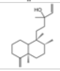	Parent ion	290.261	290.2600	3.45
DXS-GGPPS + ArTPS2 + SsSS	5524-5579	5354-5508 & 5625-5786	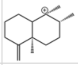	Second largest fragment (decalin core)	191.1794	191.1793	0.52
DXS-GGPPS + ArTPS2 + SsSS	5524-5579	5354-5508 & 5625-5786	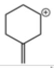	Largest fragment	95.0856	95.0854	2.10
DXS-GGPPS + TchaTPS1	5524-5579	5354-5508 & 5625-5786	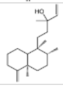	Parent ion	290.261	290.2606	1.38
DXS-GGPPS + TchaTPS1	5524-5579	5354-5508 & 5625-5786	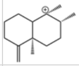	Second largest fragment (decalin core)	191.1794	191.1792	1.05
DXS-GGPPS + TchaTPS1	5524-5579	5354-5508 & 5625-5786	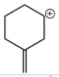	Largest fragment	95.0856	95.0854	2.10
DXS-GGPPS + TchaTPS2	5524-5579	5354-5508 & 5625-5786	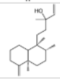	Parent ion	290.261	290.2596	4.82
DXS-GGPPS + TchaTPS2	5524-5579	5354-5508 & 5625-5786	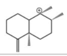	Second largest fragment (decalin core)	191.1794	191.1792	1.05
DXS-GGPPS + TchaTPS2	5524-5579	5354-5508 & 5625-5786	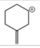	Largest fragment	95.0856	95.0854	2.10
DXS-GGPPS + TchaTPS3	5524-5579	5354-5508 & 5625-5786	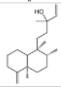	Parent ion	290.261	290.2587	7.92
DXS-GGPPS + TchaTPS3	5524-5579	5354-5508 & 5625-5786	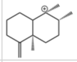	Second largest fragment (decalin core)	191.1794	191.1792	1.05
DXS-GGPPS + TchaTPS3	5524-5579	5354-5508 & 5625-5786	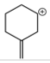	Largest fragment	95.0856	95.0854	2.10
DXS-GGPPS + TcTPS1	5524-5579	5354-5508 & 5625-5786	Picture	Parent ion	290.261	Below annotaiton threshold	NA
DXS-GGPPS + TcTPS1	5524-5579	5354-5508 & 5625-5786	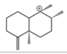	Second largest fragment (decalin core)	191.1794	191.1794	0.00
DXS-GGPPS + TcTPS1	5524-5579	5354-5508 & 5625-5786	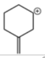	Largest fragment	95.0856	95.0855	1.05
DXS-GGPPS + ArTPS2	6830-6940	6496-6667 & 7015-7230	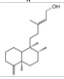	Parent ion	290.261	Below annotaiton threshold	NA
DXS-GGPPS + ArTPS2	6830-6940	5354-5508 & 5625-5787	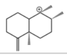	Second largest fragment (decalin core)	191.1794	191.1793	0.52
DXS-GGPPS + ArTPS2 + SsSS	6830-6940	5354-5508 & 5625-5789	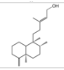	Parent ion	290.261	Below annotaiton threshold	NA
DXS-GGPPS + ArTPS2 + SsSS	6830-6940	5354-5508 & 5625-5790	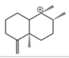	Second largest fragment (decalin core)	191.1794	191.1793	0.52
DXS-GGPPS + ArTPS2 + SsSS	6830-6940	5354-5508 & 5625-5791	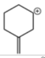	Largest fragment	95.0856	95.0855	1.05
DXS-GGPPS + TchaTPS1	6830-6940	5354-5508 & 5625-5792	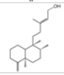	Parent ion	290.261	Below annotaiton threshold	NA
DXS-GGPPS + TchaTPS1	6830-6940	5354-5508 & 5625-5793	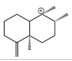	Second largest fragment (decalin core)	191.1793	191.1792	0.52
DXS-GGPPS + TchaTPS1	6830-6940	5354-5508 & 5625-5794	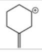	Largest fragment	95.0855	95.0854	1.05
DXS-GGPPS + TchaTPS2	6830-6940	5354-5508 & 5625-5792	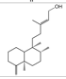	Parent ion	290.261	Below annotaiton threshold	NA
DXS-GGPPS + TchaTPS2	6830-6940	5354-5508 & 5625-5793	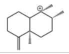	Second largest fragment (decalin core)	191.1794	191.1792	1.05
DXS-GGPPS + TchaTPS2	6830-6940	5354-5508 & 5625-5794	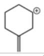	Largest fragment	95.0856	95.0854	2.10
DXS-GGPPS + TchaTPS3	6830-6940	5354-5508 & 5625-5792	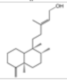	Parent ion	290.261	Below annotaiton threshold	NA
DXS-GGPPS + TchaTPS3	6830-6940	5354-5508 & 5625-5793	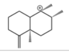	Second largest fragment (decalin core)	191.1794	191.1792	1.05
DXS-GGPPS + TchaTPS3	6830-6940	5354-5508 & 5625-5794	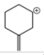	Largest fragment	95.0856	95.0854	2.10
DXS-GGPPS + TcTPS1	6830-6940	5354-5508 & 5625-5792	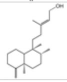	Parent ion	290.261	Below annotaiton threshold	NA
DXS-GGPPS + TcTPS1	6830-6940	5354-5508 & 5625-5793	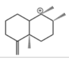	Second largest fragment (decalin core)	191.1794	191.1794	0.00
DXS-GGPPS + TcTPS1	6830-6940	5354-5508 & 5625-5794	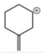	Largest fragment	95.0856	95.0855	1.05

continued from bottom.
